# Absence of CDK12 in oocyte leads to female infertility

**DOI:** 10.1038/s41419-025-07536-w

**Published:** 2025-03-27

**Authors:** Denisa Jansova, Veronika Sedmikova, Fatima J. Berro, Daria Aleshkina, Michal Dvoran, Michal Kubelka, Jitka Rezacova, Jana Rutarova, Jiri Kohoutek, Andrej Susor

**Affiliations:** 1 Laboratory of Biochemistry and Molecular Biology of Germ Cells, Institute of Animal Physiology and Genetics of the Czech Academy of Sciences, Rumburska 89, 277 21 Libechov, Czech Republic; 2 Assisted reproductive center, Institute for Mother and Child Care, Podolske nabrezi 157, Prague, Czech Republic; 3 Department of Experimental Biology, Faculty of Science, Masaryk University, 62500 Brno, Czech Republic

**Keywords:** Oogenesis, Infertility

## Abstract

Transcriptional activity and gene expression are critical for the development of mature, meiotically competent oocytes. Our study demonstrates that the absence of cyclin-dependent kinase 12 (CDK12) in oocytes leads to complete female sterility, as fully developed oocytes capable of completing meiosis I are absent from the ovaries. Mechanistically, CDK12 regulates RNA polymerase II activity in growing oocytes and ensures the maintenance of the physiological maternal transcriptome, which is essential for protein synthesis that drives further oocyte growth. Notably, CDK12-deficient growing oocytes exhibit a 71% reduction in transcriptional activity. Furthermore, impaired oocyte development disrupts folliculogenesis, leading to premature ovarian failure without terminal follicle maturation or ovulation. In conclusion, our findings identify CDK12 as a key master regulator of the oocyte transcriptional program and gene expression, indispensable for oocyte growth and female fertility.

**Figure:**
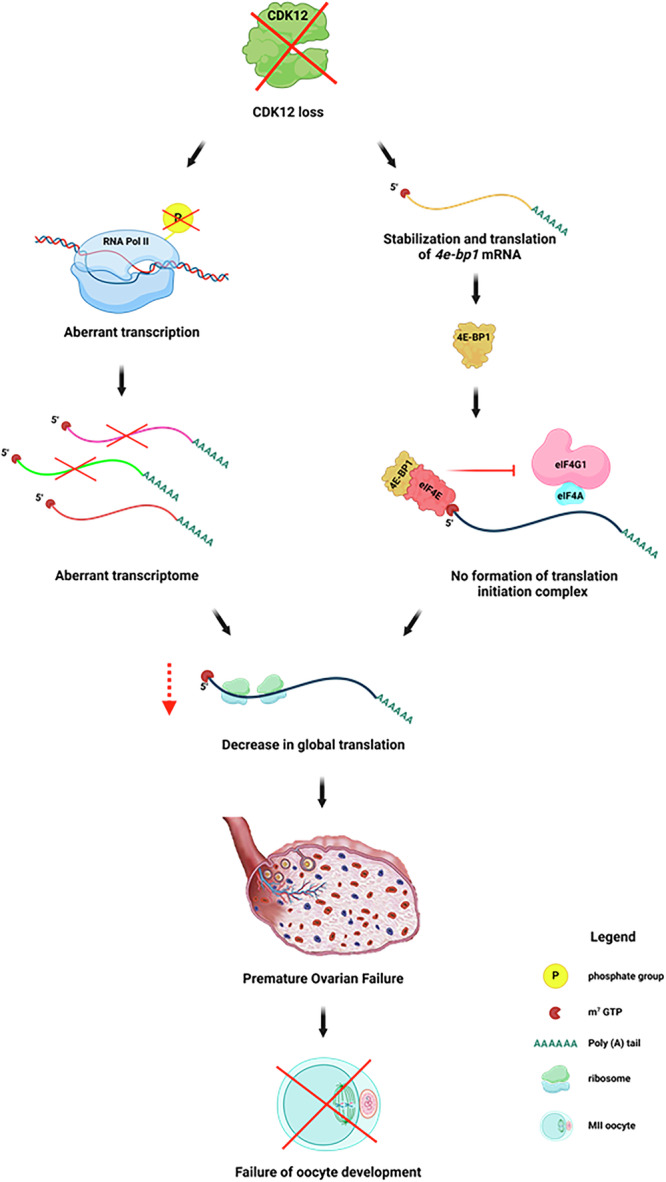
A schematic illustrating the effects of loss of CDK12 in mammalian oocytes on the regulation of transcription by polymerase II and the concomitant effects on translation. This disruption leads to an aberrant transcriptome and translatome, resulting in the absence of fully mature oocytes and ultimately female sterility.

A schematic illustrating the effects of loss of CDK12 in mammalian oocytes on the regulation of transcription by polymerase II and the concomitant effects on translation. This disruption leads to an aberrant transcriptome and translatome, resulting in the absence of fully mature oocytes and ultimately female sterility.

## Introduction

Oocytes arise from primordial germ cells and develop in follicles. The periodic activation of primordial germ cells up to the preovulatory stage is defined by oocyte growth and cytoplasmic expansion, with the synthesis and storage of maternal components such as RNA and proteins in the cytoplasm [1]. The quality of oocytes and early embryos is based on the storage of maternal components, which is regulated by various signaling pathways [2–4]. During this period, the oocyte acquires meiotic competence, i.e. the ability to resume meiosis and to enter or arrest at metaphase II [5–9], and developmental competence, the ability to support early embryonic development [10, 11].

The regulation of transcription initiation by RNA polymerase II (POLII) is of central importance for the maintenance of the oocyte and early embryonic development. Transcriptional silencing occurs in a fully-grown oocyte and continues during meiotic maturation and after fertilization in the early embryo. The hyperphosphorylated form of POLII has been found in growing oocytes, while the hypophosphorylated form is typical of mature, transcriptionally inactive GV oocytes [12]. The transcription of protein-coding genes by POLII is tightly regulated to generate proper quantities and classes of mRNA. One of the principal factors participating in the regulation of POLII activity is Cyclin-dependent kinase 12 (CDK12). Complex of CDK12 and its binding partner Cyclin K controls transcription, by phosphorylating the C-terminal domain (CTD) of the large subunit of POLII [13]. Current evidence suggests that CDK12 has a wide range of biological functions, including DNA replication [14], regulation of the expression of DNA damage response genes and cell cycle genes [15], transcription elongation [16], pre-mRNA processing [17], RNA turnover [18] and the initiation of translation of mRNA subgroups [15, 19, 20]. Blazek et al. have shown that the depletion of CDK12 leads to a reduced expression of 4% of genes [13]. CDK12 is one of the most frequently mutated genes in ovarian carcinoma [21] and these mutations lead to a loss of function [22]. Although the role of CDK12 in cancer has been broadly investigated, its specific role in oocyte development is unknown.

Based on this knowledge, we hypothesized that CDK12 is essential for maintaining the integrity of the maternal transcriptome and translatome in growing oocytes. To test this hypothesis, we generated an oocyte-specific knockout mouse model lacking CDK12 expression. Our findings revealed that the absence of CDK12 disrupts transcription by impairing POLII regulation, leading to a dysregulated maternal transcriptome and defective translation in oocytes. This dysregulation adversely affects oocyte growth, thereby impairing folliculogenesis and ultimately resulting in female infertility.

## Results

### CDK12 is essential for female fertility

**Fig. 1:**
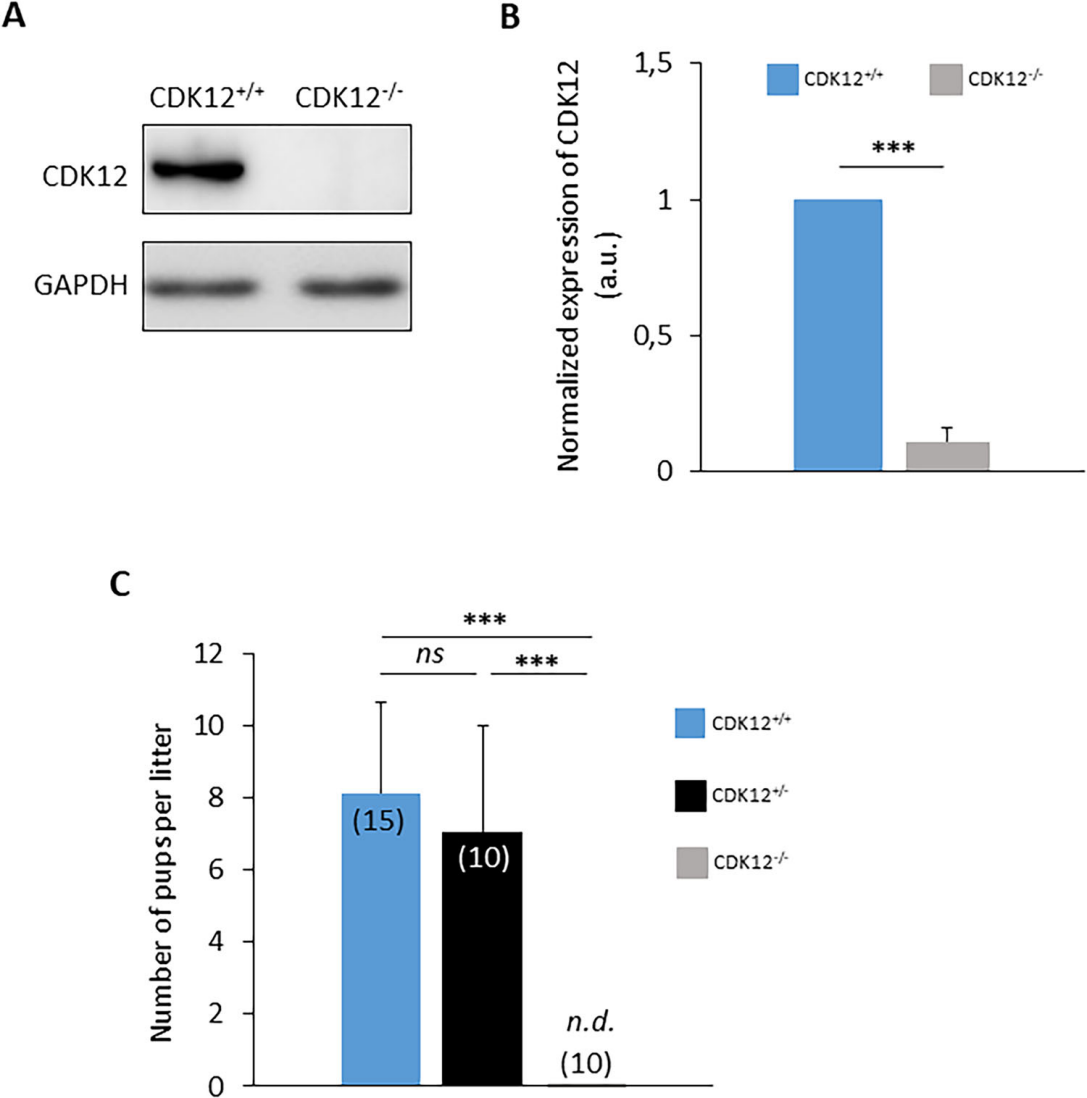
CDK12 is essential for female fertility. **A** Western blot analysis of CDK12 expression in oocytes from wild-type (CDK12^+/+^) and homozygous conditional knockout (cKO) females (CDK12^−/−^). GAPDH was used as a loading control. Data from six independent biological replicates. For *Cdk12* mRNA expression analysis, see Fig. 5. For information about conditional KO generation and CDK12 localization and expression, see SI Fig. 1. **B** Quantification of CDK12 protein expression from (**A**) normalized to GAPDH. Data are presented as mean ± SD; Student’s *t*-test: ***p < 0.001. **C** Analysis of fertility of females from different genotypes mated with proven breeder wild type males. Number of breeding pairs in parentheses. Data are presented as mean ± SE; Student’s *t*-test: *ns*, non-significant; ***p < 0.001.

To explore the requirement of Cyclin-dependent kinase 12 (CDK12) in female fertility, we performed a series of experiments using a conditional CDK12 knockout (cKO) mouse model. The aim of our study was to understand the effects of CDK12 depletion on oocyte development and overall fertility in female mice. The CDK12 cKO was generated by crossing *Cdk12*^*fx/fx*^ with a Z*p3*^*Cre*^ strain (Supporting Information 1A, B). The resulting experimental genotypes were labeled as wild-type (WT; CDK12^+/+^; Cdk12^tm1c+/+ Zp3-Cre+/+^); heterozygote (HT; CDK12^+/−^; Cdk12^tm1c+/- Zp3-Cre +/−^) and homozygote (cKO; CDK12^−/−^; Cdk12^tm1c-/- Zp3-Cre+/+^) (Supporting Information 1A, B). Immunoblot analysis confirmed the absence of CDK12 protein in the CDK12^−/−^ GV oocytes (Fig. 1A, B and Supporting Information 1C, D). In addition, immunofluorescence experiments showed that CDK12 was localized to the nucleus of WT oocytes, while it was absent in CDK12^−/−^ oocytes (Supporting Information 1C, D). Breeding experiments with CDK12^+/+^ proven breeder males showed that females lacking CDK12 in their oocytes were completely sterile, while CDK12^+/+^ and CDK12^+/−^ females were normally fertile (Fig. 1C), suggesting that there is no haploinsufficiency for CDK12.

In conclusion, the results show that the absence of maternal CDK12 in oocytes leads to female sterility.

### CDK12 is Essential to Oocyte Development and Maturation

**Fig. 2:**
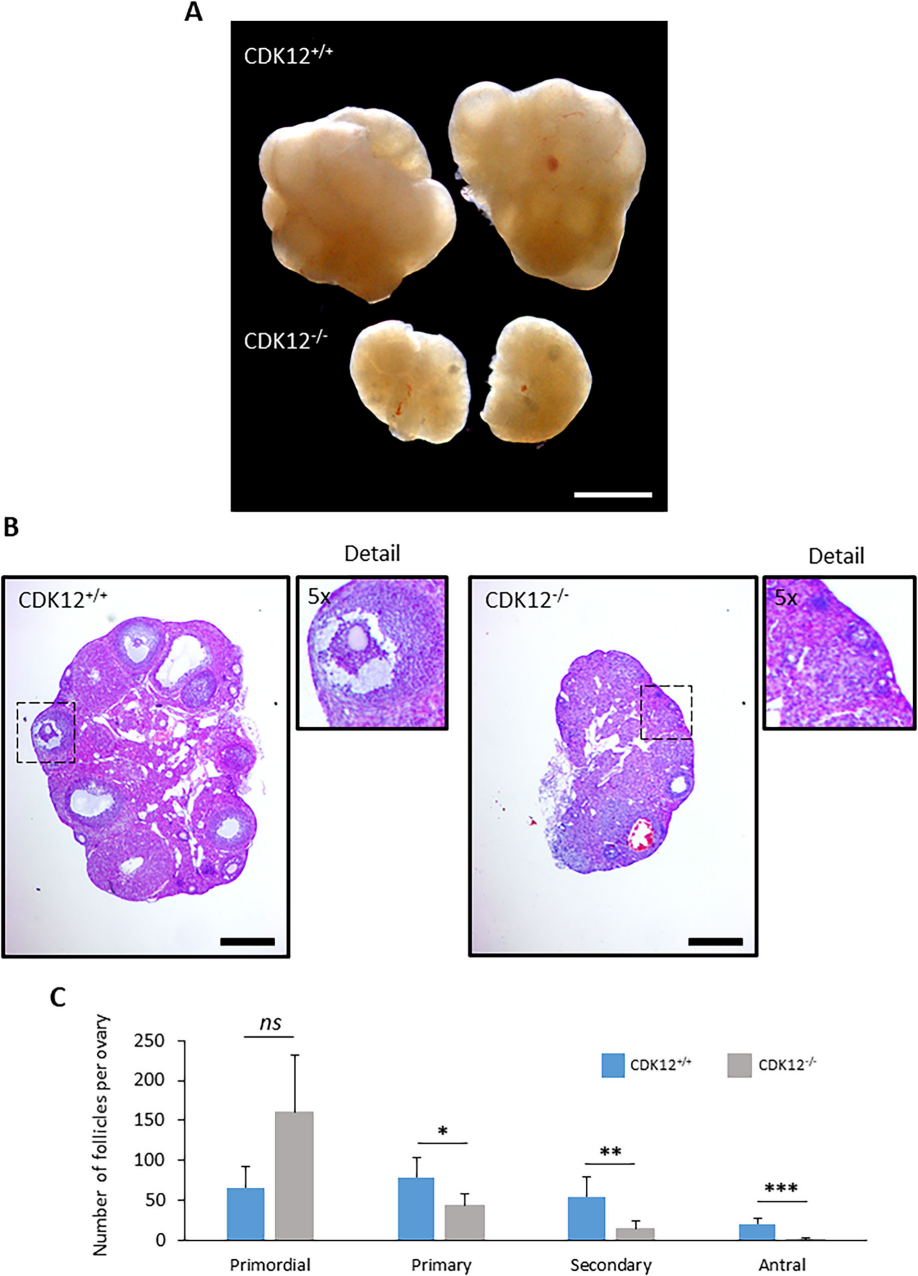
Absence of CDK12 in the oocytes leads to decreased ovarian size via ceased folliculogenesis. **A** Representative image of pair of ovaries from CDK12^+/+^ and CDK12^−/−^ mice. Scale bar 1 mm. For measurement of the size of the ovaries, see SI Fig. 2. **B** Representative images of histological sections of ovaries stained with haematoxylin and eosin. The dashed squares correspond to 5-fold magnification of the ovarian cortex, showing representative antral follicle (CDK12^+/+^) and primordial follicle (CDK12^−/−^). Data from two independent biological replicates, *n* = 6 per group; scale bars 400 µm. For depiction of the corpus luteum in the ovaries, see SI Fig. 2. **C** Quantification of follicles from CDK12^+/+^ and CDK12^−/−^ mice. Data from three females for each genotype, data are presented as mean ± SE; Student’s *t*-test: *ns*, non-significant; *p < 0.05; **p < 0.01; ***p < 0.001.

**Fig. 3:**
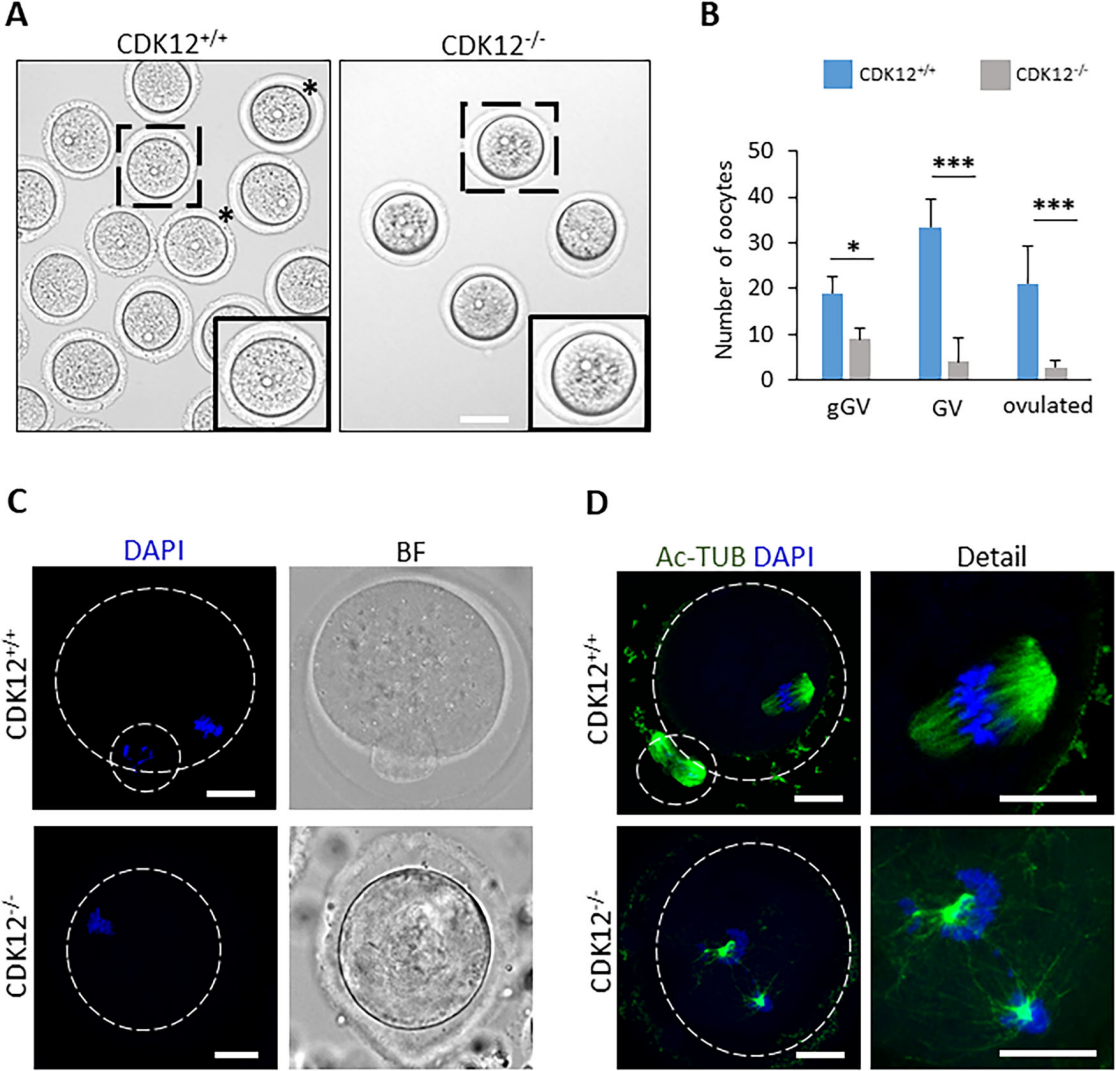
CDK12 is Essential for oocyte development and maturation. **A** Representative images of morphology of CDK12^+/+^ and CDK12^−/−^ oocytes. Dashed squares show details of oocytes, asterisks show fully grown oocytes; scale bars 60 µm. **B** Quantification of growing (gGV), fully-grown (GV) and ovulated (post hCG) oocytes isolated from ovaries. Data from five independent biological replicates. Data are presented as mean ± SE; Student’s t-test: *p < 0.05; ***p < 0.001. **C** Morphology of CDK12^+/+^ and CDK12^−/−^ ovulated oocytes. Data from three biological replicates and *n* ≤ 30 cells per group. DAPI (blue), bright field (BF); the dashed line shows the cell cortex; scale bars 20 µm. **D** Representative images of spindle morphology labeled with acetylated α-tubulin (Ac-TUB, green) in ovulated CDK12^+/+^ and CDK12^−/−^ oocytes. Data from three biological replicates and n ≤ 25 per group. Details show the enlargement of the spindle area. DAPI (blue); the dashed line shows the cell cortex; scale bars 20 µm.

Next, we investigated why absence of CDK12 in oocytes leads to female infertility. It is known that oocytes go through a series of developmental stages prior to fertilization, including oocyte growth, acquisition of meiotic competence and maturation into fertilizable MII oocyte. To understand the main cause of infertility in cKO mice, we analyzed the ovaries and oocyte quality. Following the standard procedure of superovulation, we examined the morphology of the ovaries. Despite the mice having the equal body size, the ovaries of cKO females were significantly smaller compared to those of WT females (Fig. 2A and Supporting Information 2A). Histological analysis of cKO ovaries and quantification of follicles revealed a reduced number of primary follicles and almost no antral follicles (Fig. 2B, C), resulting in the premature ovarian failure (POF) phenotype. In addition, rare ovulations lead to the formation of the corpus luteum in the cKO ovaries, which shows that the ovaries respond to hormonal stimulation and ovulation (Supporting Information 2B). Next, we found that post PMSG stimulation most of the isolated oocytes from WT females were fully grown germinal vesicle (GV) (Fig. 3A, B). In contrast, oocytes from cKO females were predominantly growing GV oocytes (Fig. 3A, B). Following the standard procedure of superovulation, we obtained only a few ovulated oocytes in the oviduct of the CKO females, although the WT genotype ovulated a normal number of oocytes (Fig. 3B). In contrast, cKO females do not produce MII oocytes during scarce ovulation (Fig. 3C, D). Instead, the ovulated CDK12^−/−^ oocytes were devoid of polar body, with disorganized chromosomes and polymerized tubulin (Fig. 3C, D).

In summary, the absence of CDK12 in the oocyte leads to an interruption of oocyte development to fully grown GV stage and thus to a termination of folliculogenesis, resulting in female infertility.

### The absence of CDK12 suppresses transcriptional activity in developing oocytes

**Fig. 4:**
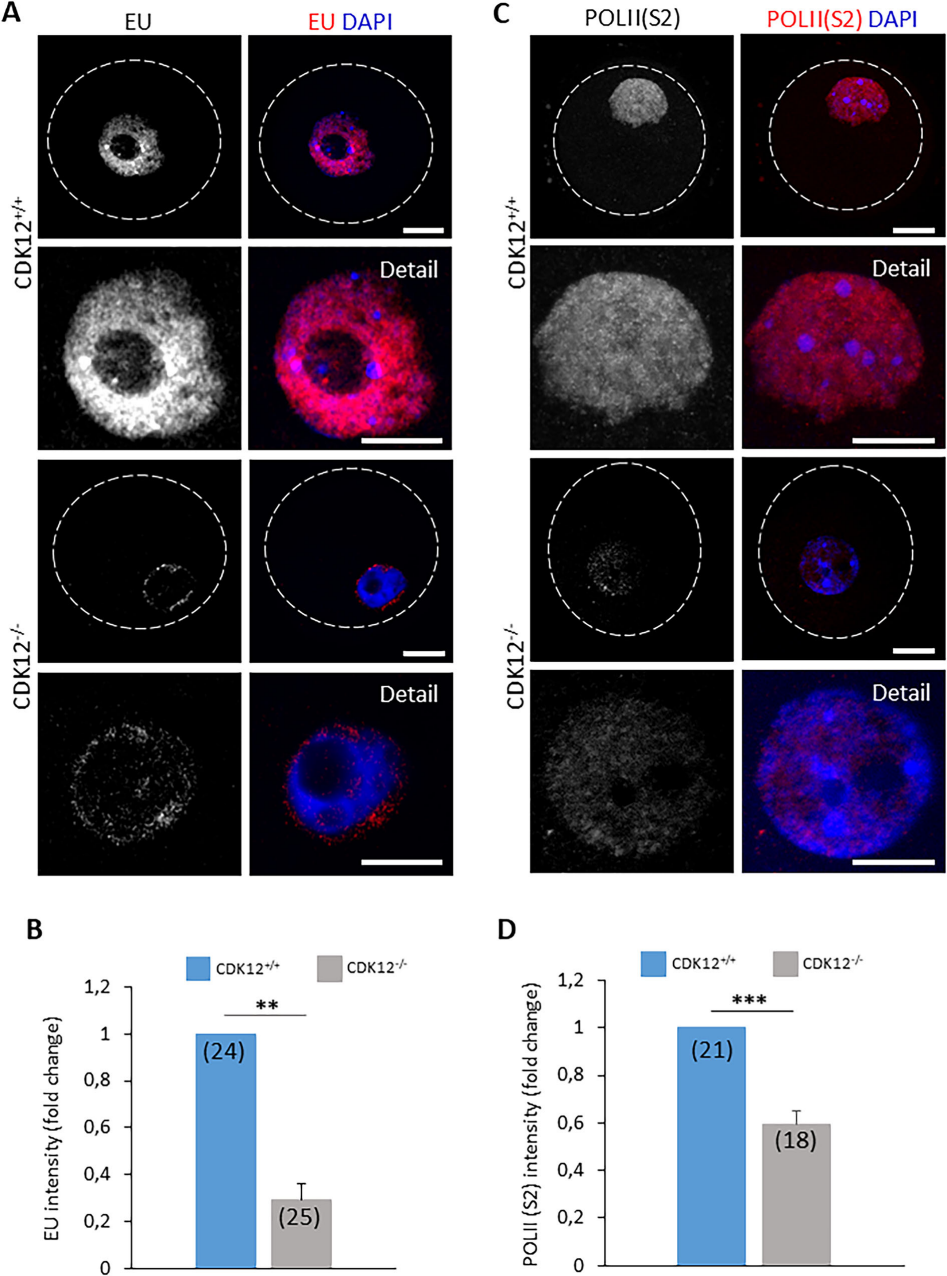
The absence of CDK12 supress transcriptional activity in developing oocytes. **A** Detection of transcriptional activity with 5-Ethynyl Uridine (EU; gray and red) in CDK12^+/+^ and CDK12^−/−^ oocytes. Data from three independent biological replicates. Details show a magnification of the nucleus; DAPI (blue); the dashed line depicts the cell cortex; scale bar 20 µm. **B** Quantification of EU fluorescence in the nucleus from (**A**). The values from CDK12^+/+^ were set as 1. The number of cells is shown in parentheses. Data are presented as mean ± SE; Student’s *t*-test: **p < 0.01. **C** Detection of phosphorylation of Polymerase II at Serine 2 (POLII(S2); gray and red) by immunocytochemistry. Data from three independent experiments. The details show an enlargement of the nuclear region. DAPI (blue); the dashed line depicts the cell cortex; scale bar 20 µm. **D** Quantification of POLII (S2) fluorescence intensity in the nucleus of CDK12^+/+^ and CDK12^−/−^ oocytes from the (**C**) experiment. The values from CDK12^+/+^ were set as 1. The number of cells is shown in parentheses. Data are presented as mean ± SE; Student’s *t*-test: ***p < 0.001.

**Fig. 5:**
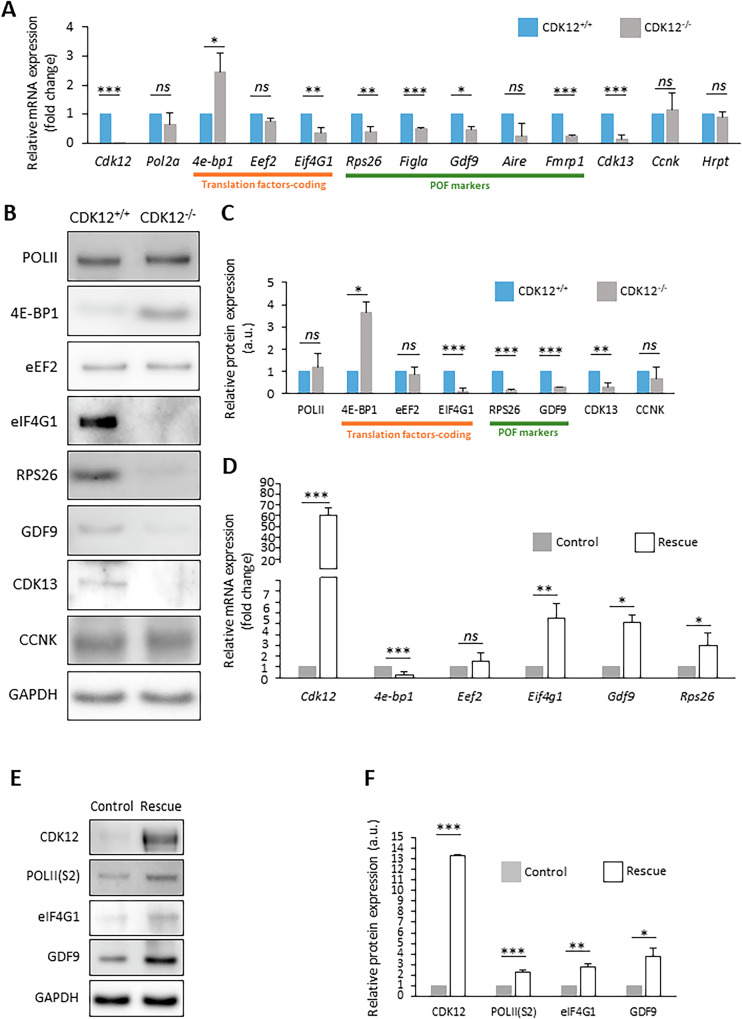
The absence of CDK12 influences the expression of a subset of mRNAs in the oocyte. **A** Quantitative RT-PCR analysis of selected mRNAs. RNAs coding for translational factors (underlined in orange) and markers for premature ovarian failure markers (underlined in green). Data normalized to *Hrpt* mRNA. Data from three independent biological replicates. Values from CDK12^+/+^ oocytes were set as 1. Data are presented as mean ± SD; Student’s *t*-test: *ns*, non-significant; *p < 0.05, **p < 0.01; ***p < 0.001. **B** Western blot analysis of candidate proteins selected from (**A**). Values from CDK12^+/+^ oocytes were set as 1. GAPDH was used as a loading control. Data from at least three independent biological replicates. For the phosphorylation of 4E-BP1, see Fig. 3. **C** Quantification of candidate proteins from (**B**). Data are presented as mean ± SE; Student’s *t*-test: *ns*, non- significant; *p < 0.05; ***p < 0.001. **D** Quantitative RT-PCR analysis of the expression of selected mRNAs in control (microinjection of *H2b-Gfp* RNA into CDK12^−/−^ oocytes; gray) and in CDK12^−/−^ oocytes microinjected with *Cdk12 m*RNA (rescue; white). Data from three independent biological replicates. Values were normalized to the number of oocytes per sample. Data are presented as mean ± SD; Student’s *t*-test: *ns*, non-significant; *p < 0.05. **E** Western blot analysis of selected proteins after CDK12 overexpression. GAPDH was used as a loading control. Data from three biological replicates. **F** Quantification of candidate proteins from (**E**). Data are presented as mean ± SE; Student’s *t*-test: *ns*, non-significant; *p < 0.05; **p < 0.01; ***p < 0.001.

Our results suggest that the main cause of infertility is impaired development of the growing oocytes. Previous reports indicated a close link between CDK12 and the regulation of transcription via phosphorylation of RNA Polymerase II (POLII) [18]. We hypothesized that this regulatory function of CDK12 is critical for proper gene expression during oocyte growth. First, we found that CDK12 is predominantly localized in the oocyte nucleus (Supporting Information Fig. 1C). In addition, proximity ligation assay shows that CDK12 is localized together with its binding partner cyclin K (CCNK) in the nucleus of the mouse and human oocyte (Supporting Information 3). To assess overall transcription, we next labeled newly synthesized RNA with 5-ethynyluridine (EU) in growing oocytes. CDK12^−/−^ oocytes exhibited a 71% decrease in EU staining compared to CDK12^+/+^ oocytes, indicating a significant decrease in global transcription (Fig. 4A, B). In addition, the active form of RNA Polymerase II (Ser2), a marker for transcription elongation, was reduced by 39% in CDK12^−/−^ oocytes (Fig. 4C, D). Importantly, there were no differences in Pol II mRNA and protein levels between groups (Fig. 5A, B).

These results suggest that the absence of CDK12 leads to an abnormal transcriptional program that impairs the formation of the oocyte mRNA reserve and consequently prevents the development to fully grown GV oocytes and the depletion of the ovarian reserve.

### The absence of CDK12 influences the expression of a subset of mRNAs in the oocyte

It is well known that oocyte development depends on proper gene expression. Given the significant reduction in transcription observed in CDK12^−/−^ oocytes (Fig. 4), we examined the expression of specific developmentally relevant classes of mRNAs encoding translational factors [15] (4E-BP1, eEF2, eIF4G1) and markers associated with premature ovarian failure [23–26] a phenotype observed in (Fig. 2) (POF; RPS26, FIGLA, GDF9, AIRE, FMRP1). Interestingly, qPCR data showed a significant 3.5-fold increase in mRNA of the translational repressor *4e-bp1* in CDK12^−/−^ oocytes (Fig. 5A). However, the mRNAs encoding the translation elongation factor EEF2 were similar, and the mRNA encoding the translation initiation factor eIF4G1 was significantly decreased in CDK12^−/−^ oocytes (Fig. 5A). In addition, all analyzed mRNAs encoding POF markers [24, 26, 27] were significantly reduced in CDK12^−/−^ oocytes (Fig. 5A). Further, we analyzed the expression of mRNAs encoding the homologous kinase CDK13 and the CDK12 binding partner CCNK (Supporting Information 3). The mRNAs of CDK12 and CDK13 were significantly decreased in CDK12^−/−^ oocytes, whereas the mRNAs of CCNK, POLII and HRPT were equally expressed in both groups (Fig. 5A).

Next, we analyzed the protein expression of selected candidate genes that had previously been examined by PCR (Fig. 5A). Immunoblotting analysis showed a positive correlation of the expression of POL II, 4E-BP1, eEF2, eIF4G1, RPS26, GDF9, CDK13 and CCNK with the corresponding mRNAs (Fig. 5B, C). To analyze the direct effect of CDK12 on the expression of candidate genes, we microinjected RNA encoding mouse CDK12 into CDK12^−/−^ oocytes. We restored the expression of CDK12 in the CDK12^−/−^ oocytes (Fig. 5D–F) and in such oocytes we observed decreased expression of the mRNA of the translational repressor *4e-bp1*. CDK12 expression increased the mRNAs of the *Eif4g1*, *Gdf9* and *Rps26* (Fig. 5D). Importantly, the presence of CDK12 in CDK12^−/−^ oocytes promoted the increase in POL II phosphorylation at serine 2, as well as the expression of eIF4G1 and the POF marker GDF9 (Fig. 5E, F).

In summary, the absence of CDK12 affects the expression of some selected mRNAs related to translation and the premature ovarian failure phenotype. Furthermore, the addition of exogenous CDK12 to CDK12^−/−^ oocytes enhances POL II activity, leading to reorganization of the expression of translation factors and markers for POF.

### Aberrant transcriptome of CDK12^−/−^ oocytes represses global translation and enhances expression of the translational repressor 4E-BP1

**Fig. 6:**
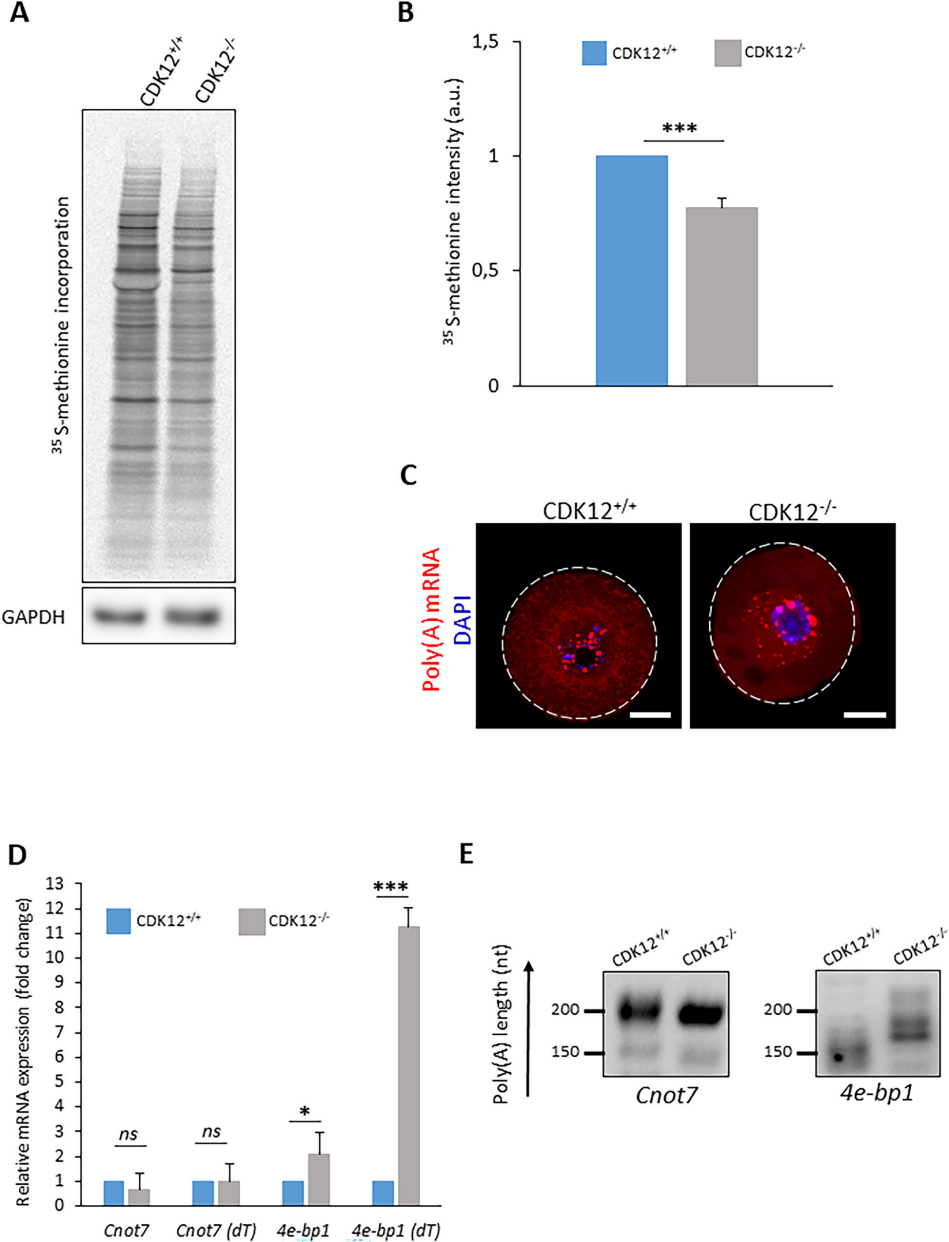
The absence of CDK12 specifically affects the stability of the mRNA encoding the translational repressor 4E-BP1. **A** Analysis of global de novo proteosynthesis by incorporation of ^35^S-methionine in CDK12^+/+^ and CDK12^−/−^ oocytes. Data from four independent biological replicates. GAPDH was used as a loading control. **B** Quantification of ^35^S-methionine incorporation from (**A**). Values from CDK12^+/+^ oocytes were set as 1. Data from four independent experiments. Data presented as mean ± SD; Student’s *t*-test: ***p < 0.001. **C** Representative confocal images of poly(A) mRNA in CDK12^+/+^ and CDK12^−/−^ oocytes. Data from three biological replicates (*n* ≥ 15). Poly(A) mRNA (red); DAPI (blue); the dashed line depicts the cell cortex; scale bars 20 µm. For the quantification of fluorescence intensity, see SI Fig. 4. **D** Quantitative RT-PCR analysis of cDNA synthetized with hexamers or oligo(dT) primers in CDK12^+/+^ and CDK12^−/−^ oocytes. Data from three biological replicates. Data are normalized to the number of oocytes. Data are presented as mean ± SD; Student’s t-test: *ns*, non-significant; *p < 0.05; ***p < 0.001. **E** Polyadenylation test (PAT) to detect the poly(A) tail length of *4e-bp1* in CDK12^+/+^ and CDK12^−/−^ oocytes. Translationally inactive *Cnot7* mRNA was used as negative control. Data from three independent biological replicates. The length of the poly(A)tail is described on the left (nt).

Considering that the absence of CDK12 promotes an aberrant maternal transcriptome and in particular, translational factors in the developing oocyte (Fig. 5A–C), we investigated, how protein synthesis was affected in CDK12^−/−^ oocytes. First, the ^35^S-methionine incorporation assay showed that CDK12^−/−^ oocytes exhibited a 23% reduction in global protein synthesis compared to WT oocytes (Fig. 6A, B). Considering the reported effect of CDK12 on RNA polyadenylation [28, 29] and the impaired transcriptome/proteome (Fig. 5A–D) in CDK12^−/−^ oocytes, the polyadenylation directly correlates with the rate of protein synthesis of mRNA. We examined global polyadenylation by RNA FISH, which showed no difference between CDK12^+/+^ and CDK12^−/−^ oocytes (Fig. 6C and Supporting Information 4). In connection to overexpression of the translational repressor 4E-BP1 and reduced translation in CDK12^−/−^ oocytes (Fig. 5A, B), we analyzed polyadenylation of the mRNA encoding 4E-BP1. The PAT assay showed a visible poly(A) shift of the 3’UTR tail of *4e-bp1* in CDK12^−/−^ oocytes, while the poly(A) tail remained unchanged in *Cnot7 mRNA*, which is translated after completion of meiosis I [30] (Fig. 6D, E).

In summary, we document that the absence of CDK12 leads to the stabilization of specific mRNAs, in particular *4e-bp1*, which is in the non-phosphorylated state (Supporting Information 5), thereby acting as a translational repressor and leading to reduced protein synthesis.

Our results clearly show that the absence of CDK12 leads to maternal infertility via the disruption of oocyte growth by a mechanism that impairs maternal transcriptome and translation (Graphical abstract). In addition, discontinued oocyte development leads to the failure of folliculogenesis, which is similar to the afollicular form of premature ovarian failure that also occurs in humans [31].

## Discussion

We have summarized here the biological function of CDK12 with a focus on female reproduction. Interestingly, the absence of CDK12 in the oocyte results in the absence of fully developed oocytes, leading to complete female sterility. With Cre, CDK12 is downregulated under the ZP3 promoter, which is activated in the growing 20 µm oocyte and reaches its maximum in the 50 µm oocyte [32]. This approach allows us to investigate the role of CDK12 in the transcriptionally active phase of oocyte development, which influences the early phase of oocyte growth and thus folliculogenesis. Although ovulation is promoted even in the absence of CDK12, it is only sparse and oocytes do not mature to a stage that can be fertilized and at least promote subfertility. Studies in mice have demonstrated that transcriptional activity and the transition of chromatin conformation from growing to fully grown GV oocyte are critical for oocyte maturation and subsequent embryo development [33–35]. Our analysis revealed a reduced number of antral follicles and an increased proportion of growing oocytes in CDK12-deficient mice. We hypothesize that the elevated proportion of growing CDK12^−/−^ oocytes results from their limited meiotic competence. A recent transcriptional profile analysis of human growing and fully grown oocytes showed that CDKs involved in transcription via POL II have higher expression in human growing oocytes, indicating the high activity characteristic of this developmental stage [35].

The CDK12/CCNK complex has been shown to be required for the promotion of transcription elongation by RNA polymerase II activation via the phosphorylation of its carboxyl-terminal domain [36]. CDK12 has sequences for nuclear localization [37]. In the oocyte, CDK12/CCNK complexes are abundant in the nucleus, supporting their biological function in transcription [13, 16]. Although CDK12 and CDK13 share a high degree of homology, they exhibit only partial redundancy in their roles in polymerase II-mediated transcription [16, 38]. In cancer cell lines, the genetic deletion or chemical inhibition of CDK12 kinase activity has virtually no effect on CDK13 protein levels, and never leads to such a dramatic downregulation seen in growing oocytes [13, 39]. It is therefore difficult to decide whether the 69% decrease in global transcription is due to the absence of CDK12 or CDK13. In cancer cell lines, inhibition of the kinase activity of both kinases by several functionally different inhibitors affected transcription efficiency by 10 - 20% [13, 38]. One can speculate that the role of CDK12 and CDK13 in regulating transcription in cancer cell lines is different from that in primary cells such as oocytes. Not surprisingly, the decrease in global transcription was accompanied by a 39% decrease in the Ser2 phosphorylation detecting of elongating Pol II. Since we could still detect Pol II phosphorylated at Ser in the nucleus, it is very likely that CDK9 is the kinase responsible for the remaining Pol II(Ser2) signal, since CDK9 is active in the GVs of porcine oocytes [40]. Furthermore, the chemical inhibition of CDK12, CDK13 or both together affected up to 15% of all genes, clearly indicating that the transcriptional program in growing oocytes is mainly the transcription of specialized and developmentally relevant genes [38]. Although we did not perform a global transcriptome analysis, we clearly found decreased mRNA for POF markers and translation factors with the sole exception of the *4e-bp1* mRNA. Suggesting that the absence of CDK12 (i) promotes the transcription of specific genes, (ii) impairs a specific transcriptional program during the activation of the ZP3 promoter in the oocyte, and/or (iii) indirectly affects transcription by altering the expression of factors that negatively modulate transcription. Our results show that the absence of CDK12 affects the transcriptome of the growing oocyte by interfering with the activity of Pol II, which in turn affects translation. Previous reports showed that the yeast ortholog of CDK12, Ctk1 [16, 41] stimulates mRNA translation globally [20, 42]. Similarly, we found an additional role of CDK12 that involves the stabilization of specific mRNA with increased polyadenylation, thereby promoting their translation. Indeed, CDK12 has been identified as a nuclear co-transcriptional polyadenylation factor that either ensures the processing of the 3’-end through a cleavage and polyadenylation mechanism, or blocks the premature cleavage and polyadenylation (PCPA) of hnRNA [29, 43]. Apart from the effect of CDK12 on the polyadenylation of 4E-BP1 mRNA, the deletion of CDK12 resulted in a moderate increase in the protein level of the 5’-cap-binding mRNA repressor 4E-BP1 in CDK12^−/−^ oocytes. Our previously published results provided evidence that expression of the inactive translational repressor 4E-BP1 leads to aberrant proteosynthesis in fully developed, transcriptionally silent oocyte [44]. Most mRNAs accumulated during oocyte growth are translationally repressed and are translated later when transcription is repressed [45, 46]. Therefore, 4E-BP1 may play a key role in mRNA storage process, and the overexpression of 4E-BP1 observed in CDK12^−/−^ oocytes suppresses the translation of already abnormally expressed mRNAs, the translation of which promotes oocyte growth. We previously, reported that inactivation of 4E-BP1 occurs at later stages of oocyte development, after entry into the M phase [44], and similarly, CDK12 phosphorylates/inactivates 4E-BP1 and promotes the translation of specific mRNA of factors involved in mitotic spindle regulation and chromosome segregation [15]. Importantly, the translational repressor 4E-BP1 is inactivated by phosphorylation during the metaphase transition in cells and oocytes [15, 44]. The unphosphorylated form of 4E-BP1 is therefore present in growing GV oocytes. Nevertheless, we still do not know whether and how the mRNA stabilization and translation of 4E-BP1 is directly or indirectly influenced by CDK12. In addition, the reduced expression of the translation initiation factor eIF4G1 and aberrant transcriptome in CDK12^−/−^ oocytes, likely contribute to reduced global translation.

CDK12 is involved in the DNA-damage repair (DDR) pathway by regulating the expression of several DDR machinery components, including BRCA1, ATM, ATR, and Fanconi anemia (FANC) genes [13]. CDK12-deficient blastocysts exhibit abnormal morphology and developmental failure due to increased DNA damage within the inner cell mass [47]. Beyond its impact on transcription and translation in growing oocytes, the loss of CDK12 likely impairs the expression of DDR genes. Recent studies identifying factors responsible for primary and premature ovarian insufficiency (POI) have reported either dramatic downregulation or non-functional variants of several DDR pathway members, such as BRCA1, RAD51, ATR, MSH4, MSH5, and FANC genes [23, 48].

Long-term depletion of CDK12 leads to cell cycle arrest in the G2/M phase [27]. Moreover, novel deleterious mutations in the kinase domain of CDK12 (H857Y/R, F8787S, T893I) have been identified in ovarian cancer patients [49]. These mutations mimic a loss-of-function phenotype, although their specific impact on fertility remains unknown. Interestingly, patients with CDK13-related disorders exhibit pregnancy complications in 29.4% of cases [50–53].

Among the downregulated transcripts and proteins, premature ovarian failure (POF) markers such as *Rps26*, *Figla*, *Gdf9*, *Aire* and *Fmrp1* [24, 25, 54–61] were detected, so the absence of CDK12 clearly resembles a POF phenotype [62–64]. We have compiled a list of genes linked to ovarian development and function in both human and mouse, which are regulated by CDK12 (Supplementary Table 1) [57, 65–67]. POF is a clinical disorder characterized by hypogonadism and amenorrhea, and affects 1–3% of women under 40 years of age [68–71]. The overall prevalence of familial POF ranges from 4% to 31% [70]. The appearance of the ovaries varies from the complete depletion of follicles to the presence of a variable population of follicles that fail to develop [68, 72]. The pregnancy rate among women with POF ranges from 2.2% to 14.2% [73]. GDF9 and RPS26 have been described as important factors for oocyte growth and the recruitment of a primordial follicle into the pool of growing follicles [25, 74]. GDF9, a protein secreted from the oocyte into the follicle, influences the proliferation, differentiation, steroid hormone synthesis, apoptosis and cumulus expansion of granulosa cells [75]. GDF9 levels increase dramatically with oocyte growth during preantral folliculogenesis and remain high until ovulation [74] in oocytes of various mammalian species, including humans [76], sheep [77], bovines [77] and rats [78], indicating the universality of the role of GDF9. In addition, CDK12 has been associated with follicular atresia (reduction in the number of ovulating follicles) and early menopause in humans [62, 64, 79]. CDK12 has been also shown to play a role in the PI3K/AKT/mTOR pathway, which is a critical signaling cascade involved in primary ovarian insufficiency (POF) [15, 65, 66, 80, 81].

Taken together, our results clearly demonstrate that the absence of CDK12 leads to an abnormal transcriptome and translatome of the growing oocyte, which in turn results in the absence of a fully mature oocyte and leads to female infertility. In addition, we provide insight into the etiology of premature ovarian failure, in which CDK12 may also play an important role in humans, warranting further investigation.

## Methods

### Oocyte isolation and cultivation

Experimental genotypes were designated as wild-type (WT; CDK12^+/+^; Cdk12^tm1c+/+^ Zp3-Cre^+/+^) and homozygotes (cKO; CDK12^−/−^; Cdk12^tm1c−/−^ Zp3^+/+^). Oocytes were obtained from C57BL/6 J mice that were at least 5 weeks of age. Females were stimulated with 5 IU of pregnant mare serum gonadotropin (PMSG; Folligon; Merck Animal Health) per mouse 46 h prior to oocyte isolation. To obtain MII, the mice were primed with 5 IU human chorionic gonadotropin (hCG, Pregnyl, N.V. Organon). Oocytes were isolated in transfer medium (TM) supplemented with 100 µM 3-isobutyl-1-methylxanthine (IBMX, Sigma Aldrich) to prevent the spontaneous resumption of meiosis. Selected oocytes were denuded and cultured in M16 medium (Millipore) without IBMX at 37 °C, 5% CO_2_ for 0 h (GV) or 12 h (MII) [82]. All animal experiments were performed in accordance with the guidelines and protocols approved for the Laboratory of Biochemistry and Molecular Biology of Germ Cells at the Institute of Animal Physiology and Genetics in the Czech Republic. All animal experiments were conducted in accordance with Act No. 246/1992 on the Protection of Animals from Cruelty, issued by the Ministry of Agriculture under the number 67756/2020MZE-18134. Human oocytes not used for human reproduction were obtained from the Institute for the Care of Mother and Child in Prague. The project was approved and accredited by the Ethics Committee of the Institute for the Care of Mother and Child (#1/3/4/2022; NU-23-07-0005). All patients gave informed consent to the use of their immature oocyte(s) this study.

### Animals

Cdk12^fx/fx^ mice on a C57BL/6 J genomic background were provided by Jiri Kohoutek. Cdk12^fx/fx^ mice were crossed with Zp3-Cre mice to generate the oocyte-specific Cdk12 knockout (CDK12^−/−^). The target construct contains loxP sites in the 3 and 4^th^ exon (8 nucleotides in the defective 4th exon and a stop codon inserted into the open reading frame). The primers used for PCR to genotype Cdk12^fx/fx^ were primer mCDK12 Flx-F–CTTCAGACAGTGTCAGACCACCTGGAGAAGC; primer mCDK12 Y3-R–CCTCTGACCTCCCAATGTGTGCATGACAC; F-ZP3-GGTGGAGAATGTTAATC and R-ZP3-TATTCGGATCATCAGCTA. All experiments were performed according to the guidelines and with the approval of Institutional Animal Care. Pairs of 8-week-old female mice of genotypes CDK12^+/+^, CDK12^+/−^ and CDK12^−/−^ were continuously mated with proven CDK12^+/+^ males to test the fertility of the females. The number of pups was recorded over a period of 8 months.

### Histology of ovaries

Ovaries were fixed in 4% PFA solution (Sigma) for 48 h, then placed in 70% ethanol solution and subsequently placed in labeled histological cassettes. Samples were processed using an automated tissue processor (Leica ASP 6025, Leica Microsystems, Germany) and embedded in paraffin blocks using a Leica EG 1150H paraffin embedding station (Leica Microsystems, Germany). Sections of 3 μm were cut with a microtome (Leica RM2255, Leica Microsystems, Germany) on standard glass slides (Waldemar Knittel, GmbH, Germany), every 10th section was collected, 3–12 sections were collected per slide. Slides were stained with hematoxylin–eosin and mounted using a Leica ST5020 automated stainer in combination with a Leica CV5030 mounting frame. The number of follicles was quantified using a stereomicroscope (Zeiss Stemi 2000, Germany). The analysis of ovarian follicles was performed by two different investigators.

### Oocyte microinjection

Experimental oocytes Cdk12^−/−^ were injected with an in vitro prepared *Cdk12* and *H2b-Gfp* RNAs diluted to a final concentration of 20 ng/µl, the control samples were injected with *H2b-Gfp* mRNA alone. Subsequently, the injected oocytes were incubated in IBMX at 37 °C and 5% CO_2_ for 18 h. The oocytes were washed in PVA/PBS and frozen at −80 °C.

### Measurement of Overall Protein Synthesis

To measure total protein synthesis, 50 mCi of ^35^S-methionine (Perkin Elmer) was added to methionine-free culture medium, for 1 h and then oocytes were lysed in SDS-buffer and subjected to SDS–polyacrylamide gel electrophoresis. The labeled proteins were visualized by autoradiography with BasReader (FujiFilm). GAPDH was used as a loading control.

### 5-ethynyl uridine transcription assay

The 5-EU was added to the M16 medium with IBMX and incubated overnight with growing GV oocytes. Oocytes were then fixed in 4% paraformaldehyde/PBS for 15 min, permeabilized with 0.1% Triton X-100/PBS for 10 min at room temperature, and incubated for 1 h at room temperature in the dark with the Click-iT reaction cocktail (according to the manufacturer’s instructions using Alexa A555 azide (ThermoFisher, A20012) and a commercial kit (Click-iT, ThermoFisher, C10276). After incubation, oocytes were washed once with PBS and mounted on slides using DAPI in the presence of the anti-fade reagent Vectashield (H-1500, Vector laboratories). Images were captured using a confocal laser scanning microscope (Leica SP5, Leica Microsystems, Wetzlar, Germany). Images were quantified and compiled using FIJI software (version 1.8.0_172).

### RNA isolation and RT-PCR

TRIzol reagent (Invitrogen) was used for RNA extraction according to the manufacturer’s instructions. Reverse transcription was performed with a qPCRBIO cDNA Synthesis Kit (PCR Biosystems). qPCR was then carried out using QuantStudio 3 (Applied Biosystems) and Luna® Universal qPCR Master Mix (New England BioLabs) according to the manufacturer’s protocols with an annealing temperature of 60 °C. The primers are listed in Supplementary Table 2.

### Poly-A-tail length assay (PAT)

To obtain the total length of the poly(A) tail of each transcript, total RNA was extracted using the phenol-chloroform method according to the laboratory protocol. Elution was performed in 10 µl of water per sample. The isolated RNA was incubated with 1 µl of 20 mM oligo-dT annealing per sample for 5 min at 65 °C. Ligation was then carried out for 30 min at 42 °C. The ligation mix was prepared from the following components: T4 ligase (1 µl), Superscript IV 5x buffer (5 µl), 20U/µl RNAse inhibitor (1 µl), 10 mM dNTP (1 µl), 10 mM ATP (1 µl), 1 M MgCl2 (0.1 µl), 0.1 M DTT (2 µl), RNAse-free water (2 µl). The cDNA synthesis was performed by adding 1 µl Superscript II Reverse Transcriptase with the following setup: 45 min at 45 °C, 10 min at 80 °C, hold at 4 °C. The prepared cDNA was subjected to PCR with gene-specific forward primers and anchoring reverse primer (Supplementary Table 2). PCR was performed with PPP Master Mix (Top-Bio) under the following conditions: 1 min at 95 °C, 35× (30 s at 95 °C, 20 s at 55 °C, 45 s at 72 °C). PCR products were analyzed on a 1.5% agarose gel stained with GelRed (41003, Biotinum) and run at 90 V for 45 min. The gels were detected with an Azure 600 Imager (Azure Biosystems).

### RNA FISH of poly(A) mRNA

Oocytes were fixed in 4% paraformaldehyde for 15 min and permeabilized by protease III treatment (Biosearch Technologies). Samples were washed in wash buffer A (Biosearch Technologies) and incubated overnight at 42 °C in hybridization buffer (Biosearch Technologies) containing 75 nM oligo-d(T) probe with CalFluorRed635 (Biotech Generi). Samples were washed twice in wash buffer A and twice in 2× SSC (Sigma Aldrich). Samples were embedded in VectaShield medium with DAPI (H-1500, Vector Laboratories). A confocal laser scanning microscope was used for imaging (Leica SP5, Leica Microsystems, Wetzlar, Germany). Data from three biological replicates. The quantification of fluorescence intensity between CDK12^+/+^ and CDK12^−/−^ oocytes was performed using FIJI software (version 1.8.0_172).

### Immunofluorescence

Fixed oocytes (15 min in 4% PFA, Sigma Aldrich) were permeabilized for 10 min in 0.1% Triton X-100, washed in PBS with polyvinyl alcohol (PVA, Sigma Aldrich), and incubated overnight at 4 °C with primary antibodies (Supplementary Table 2) diluted in PVA/PBS. Immunofluorescence analysis was performed according to a published protocol [83]. Image quantification and compilation was performed using FIJI software (version 1.8.0_172).

### In situ proximity ligation assay (PLA)

The proximity ligation assay was performed according to the instructions of Naveni Triflex (Navinci). Oocytes were fixed in 4% PFA for 15 min and permeabilized with 0.5% TritonX/PBS for 10 min. Oocytes were washed with TBS-T solution and then transferred into primary antibodies (CCNK and CDK12, Supplementary Table 2) 1.5: 100 dilution 1TF overnight at 4 °C. The oocytes were washed in TBS-T for 15 min. The oocytes were incubated with Navenibody MTF and Navenibody RTF 1:10 dilution 2 for 1 h at 37 °C on a hot plate. The oocytes were washed every 15 min with TBS-T solution. A total of 40 µl of amplification reaction 1 was mixed according to the manufacturer’s instructions, then added to the oocytes and incubated at 37 °C for 30 min. It was then washed with TBS-T solution for 5 min. Reaction 2 was mixed according to the manufacturer’s instructions. The oocytes were incubated in 40 µl of reaction 2, in a dish protected from light, for 1 h at 37 °C. Samples were washed for 5 min and then mounted on a concave-bottomed slide (cat. # 1216492, Marienfeld,) using Vectashield mounting medium with DAPI (H-1500, Vector Laboratories). A confocal laser scanning microscope was used for the images (Stellaris8).

### Western Blotting

Oocyte lysates were analyzed on a 4–12% gradient acrylamide gel. Samples were transferred to a polyvinylidene fluoride membrane (Immobilon P; Merckmillipore) using a blotting system (Biometra GmbH) at 5 mA/cm^2^ for 25 min. The membranes were blocked for 1 h at room temperature and then incubated overnight at 4 °C with the primary antibodies listed in Supplementary Table 2. Membranes were incubated with secondary antibodies for 1 h at room temperature. Proteins were visualised by chemiluminescence using ECL (Amersham) and imaged in an Azure 600 Imager (Azure Biosystems), and the acquired signals were quantified using ImageJ (http://rsbweb.nih.gov/ij/). Data from at least three biological replicates. To detect the phosphorylation shift, oocytes were dissolved in 20 µl of 1× NEB buffer containing 800 U of LPP enzyme (P0753, New England BioLabs) and incubated at 30 °C for 1 h.

### Statistical analysis and data visualization

GraphPad Prism 8.3 was used for the statistical analysis. The statistical analysis included Student’s t-tests to determine statistical significance between groups (labeled with an asterisk). *p < 0.05; **p < 0.01, and ***p < 0.001. Mean and standard error values were calculated in MS Excel (Microsoft).

## Supplementary information

Supplementary Table 1

Supplementary Table 2

Figures and Legends SI

## Data Availability

All data generated or analyzed during this study are included in this published article and its supplementary information files.
