# Supplementary material for: Absence of CDK12 in oocyte leads to female infertility
Source: Cell Death Dis. 2025 Mar 27;16(1):213. doi: 10.1038/s41419-025-07536-w (PMC11950339; doi:10.1038/s41419-025-07536-w)
Supplement: Supplementary file 1 — Supplementary Table 1 [file 41419_2025_7536_MOESM1_ESM.pptx]

## Slide 1
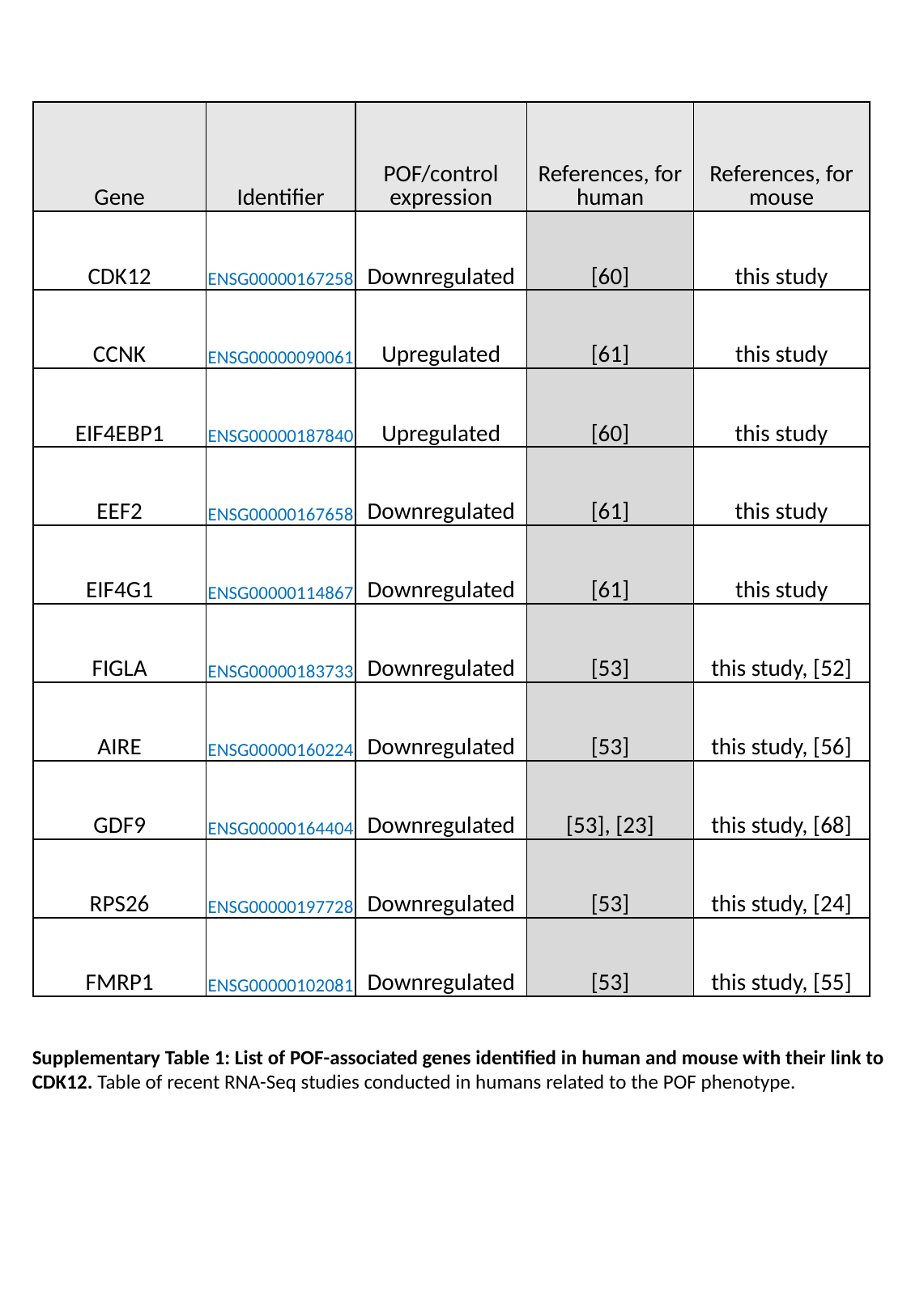

| Gene | Identifier | POF/control expression | References, for human | References, for mouse |
| --- | --- | --- | --- | --- |
| CDK12 | ENSG00000167258 | Downregulated | [60] | this study |
| CCNK | ENSG00000090061 | Upregulated | [61] | this study |
| EIF4EBP1 | ENSG00000187840 | Upregulated | [60] | this study |
| EEF2 | ENSG00000167658 | Downregulated | [61] | this study |
| EIF4G1 | ENSG00000114867 | Downregulated | [61] | this study |
| FIGLA | ENSG00000183733 | Downregulated | [53] | this study, [52] |
| AIRE | ENSG00000160224 | Downregulated | [53] | this study, [56] |
| GDF9 | ENSG00000164404 | Downregulated | [53], [23] | this study, [68] |
| RPS26 | ENSG00000197728 | Downregulated | [53] | this study, [24] |
| FMRP1 | ENSG00000102081 | Downregulated | [53] | this study, [55] |
Supplementary Table 1: List of POF-associated genes identified in human and mouse with their link to CDK12. Table of recent RNA-Seq studies conducted in humans related to the POF phenotype.
