# Supplementary material for: Absence of CDK12 in oocyte leads to female infertility
Source: Cell Death Dis. 2025 Mar 27;16(1):213. doi: 10.1038/s41419-025-07536-w (PMC11950339; doi:10.1038/s41419-025-07536-w)
Supplement: Supplementary file 2 — Supplementary Table 2 [file 41419_2025_7536_MOESM2_ESM.pptx]

## Slide 1
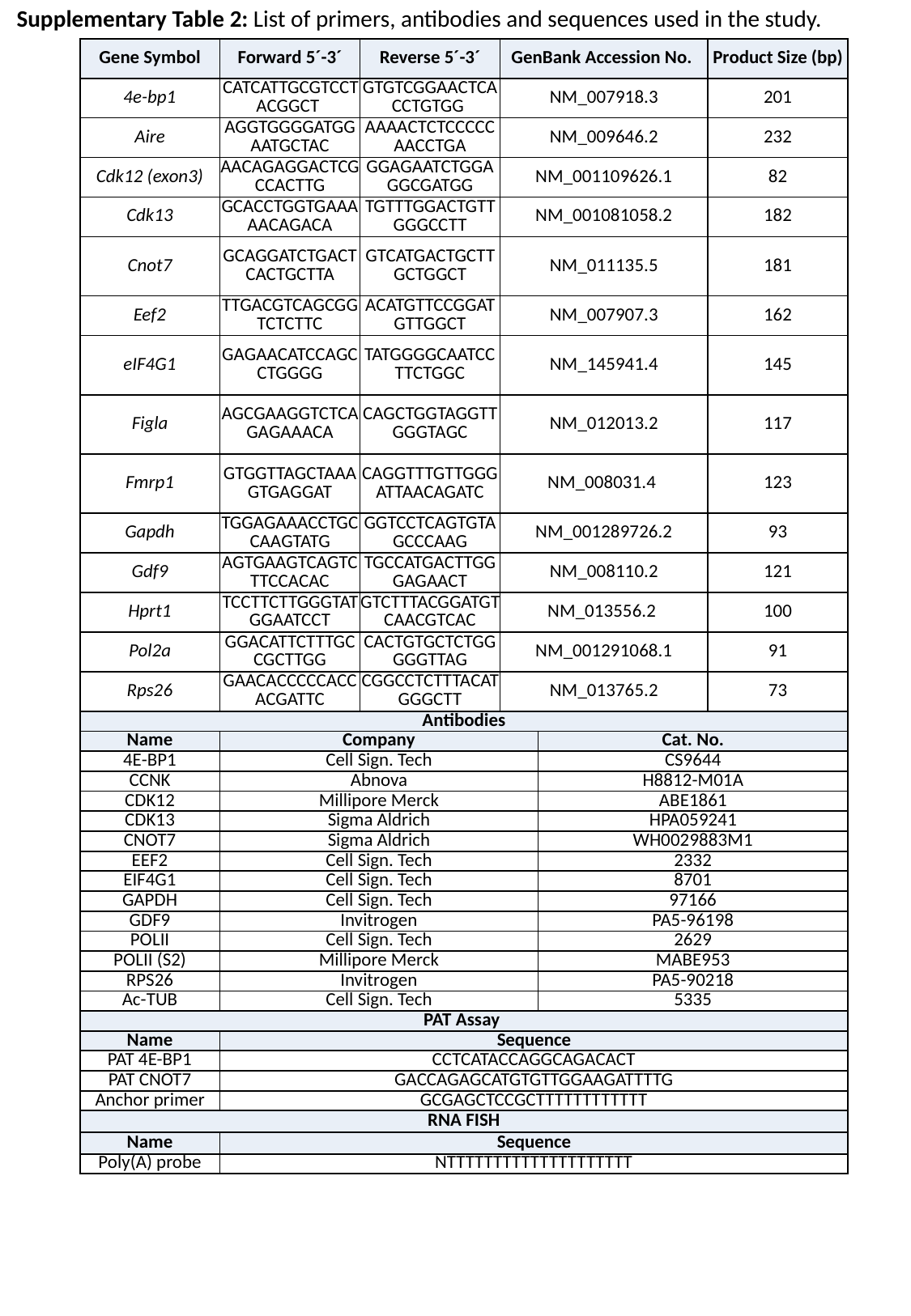

Supplementary Table 2: List of primers, antibodies and sequences used in the study.
| Gene Symbol | Forward 5´-3´ | Reverse 5´-3´ | GenBank Accession No. | | Product Size (bp) |
| --- | --- | --- | --- | --- | --- |
| 4e-bp1 | CATCATTGCGTCCTACGGCT | GTGTCGGAACTCACCTGTGG | NM\_007918.3 | | 201 |
| Aire | AGGTGGGGATGGAATGCTAC | AAAACTCTCCCCCAACCTGA | NM\_009646.2 | | 232 |
| Cdk12 (exon3) | AACAGAGGACTCGCCACTTG | GGAGAATCTGGAGGCGATGG | NM\_001109626.1 | | 82 |
| Cdk13 | GCACCTGGTGAAAAACAGACA | TGTTTGGACTGTTGGGCCTT | NM\_001081058.2 | | 182 |
| Cnot7 | GCAGGATCTGACTCACTGCTTA | GTCATGACTGCTTGCTGGCT | NM\_011135.5 | | 181 |
| Eef2 | TTGACGTCAGCGGTCTCTTC | ACATGTTCCGGATGTTGGCT | NM\_007907.3 | | 162 |
| eIF4G1 | GAGAACATCCAGCCTGGGG | TATGGGGCAATCCTTCTGGC | NM\_145941.4 | | 145 |
| Figla | AGCGAAGGTCTCAGAGAAACA | CAGCTGGTAGGTTGGGTAGC | NM\_012013.2 | | 117 |
| Fmrp1 | GTGGTTAGCTAAAGTGAGGAT | CAGGTTTGTTGGGATTAACAGATC | NM\_008031.4 | | 123 |
| Gapdh | TGGAGAAACCTGCCAAGTATG | GGTCCTCAGTGTAGCCCAAG | NM\_001289726.2 | | 93 |
| Gdf9 | AGTGAAGTCAGTCTTCCACAC | TGCCATGACTTGGGAGAACT | NM\_008110.2 | | 121 |
| Hprt1 | TCCTTCTTGGGTATGGAATCCT | GTCTTTACGGATGTCAACGTCAC | NM\_013556.2 | | 100 |
| Pol2a | GGACATTCTTTGCCGCTTGG | CACTGTGCTCTGGGGGTTAG | NM\_001291068.1 | | 91 |
| Rps26 | GAACACCCCCACCACGATTC | CGGCCTCTTTACATGGGCTT | NM\_013765.2 | | 73 |
| Antibodies | | | | | |
| Name | Company | | | Cat. No. | |
| 4E-BP1 | Cell Sign. Tech | | | CS9644 | |
| CCNK | Abnova | | | H8812-M01A | |
| CDK12 | Millipore Merck | | | ABE1861 | |
| CDK13 | Sigma Aldrich | | | HPA059241 | |
| CNOT7 | Sigma Aldrich | | | WH0029883M1 | |
| EEF2 | Cell Sign. Tech | | | 2332 | |
| EIF4G1 | Cell Sign. Tech | | | 8701 | |
| GAPDH | Cell Sign. Tech | | | 97166 | |
| GDF9 | Invitrogen | | | PA5-96198 | |
| POLII | Cell Sign. Tech | | | 2629 | |
| POLII (S2) | Millipore Merck | | | MABE953 | |
| RPS26 | Invitrogen | | | PA5-90218 | |
| Ac-TUB | Cell Sign. Tech | | | 5335 | |
| PAT Assay | | | | | |
| Name | Sequence | | | | |
| PAT 4E-BP1 | CCTCATACCAGGCAGACACT | | | | |
| PAT CNOT7 | GACCAGAGCATGTGTTGGAAGATTTTG | | | | |
| Anchor primer | GCGAGCTCCGCTTTTTTTTTTTT | | | | |
| RNA FISH | | | | | |
| Name | Sequence | | | | |
| Poly(A) probe | NTTTTTTTTTTTTTTTTTTTT | | | | |
