# Supplementary material for: Absence of CDK12 in oocyte leads to female infertility
Source: Cell Death Dis. 2025 Mar 27;16(1):213. doi: 10.1038/s41419-025-07536-w (PMC11950339; doi:10.1038/s41419-025-07536-w)

SI Fig. 1

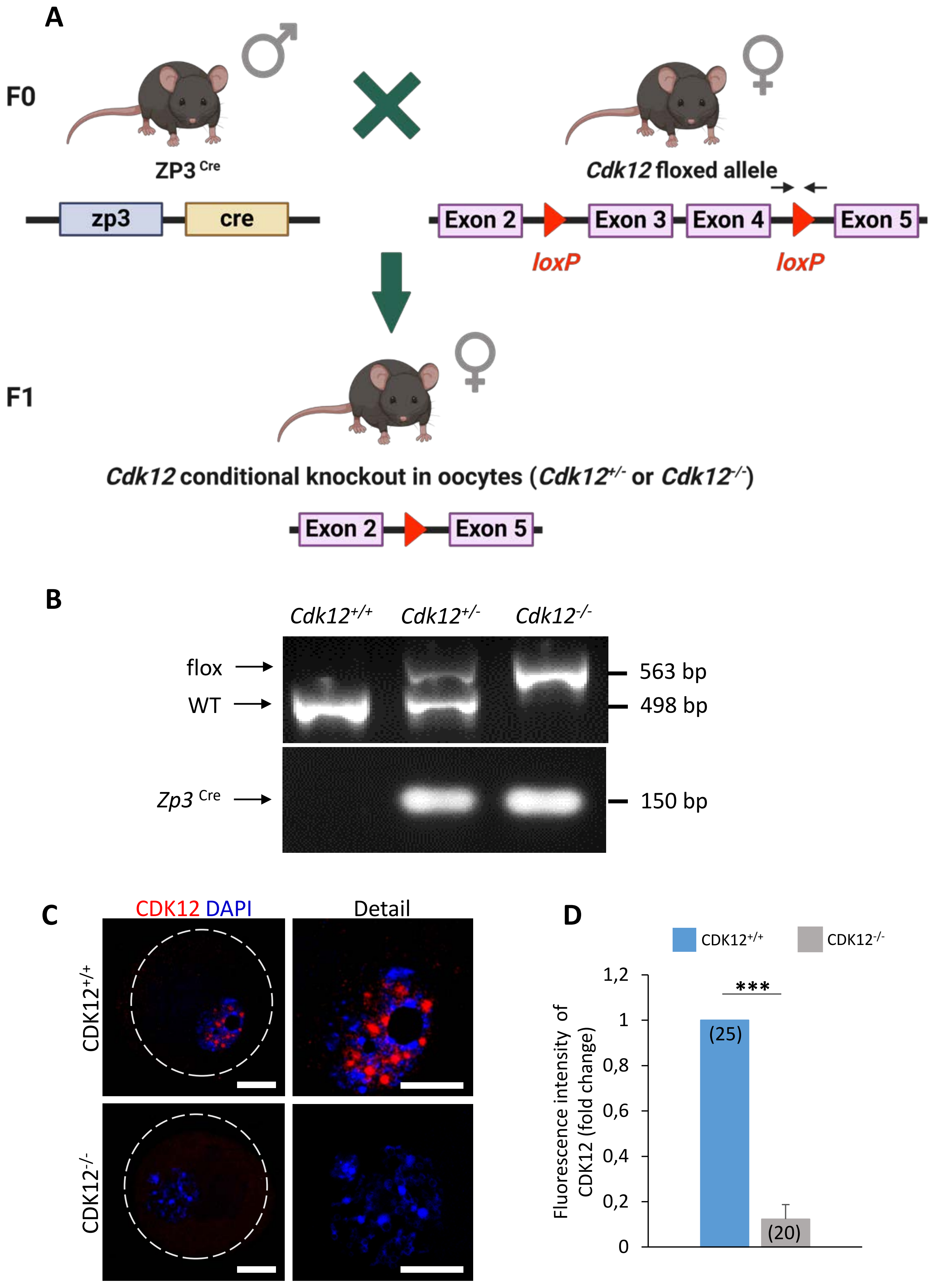
A
F0
ZP3 Cre
zp3
cre
Cdk12 floxed allele
Exon 2
Exon 3
Exon 4
Exon 5
loxP
loxP
F1
Cdk12 conditional knockout in oocytes (Cdk12+/- or Cdk12-/-)
Exon 2
Exon 5
B
Cdk12+/+
Cdk12+/-
Cdk12-/-
flox
WT
563 bp
498 bp
Zp3 Cre
150 bp
C
CDK12 DAPI
Detail
CDK12+/+
CDK12-/-
D
CDK12+/+
CDK12-/-
Fluorescence intensity of CDK12 (fold change)
1,2
1
0,8
0,6
0,4
0,2
0
***
(25)
(20)

SI Fig. 1: The generation of mice with conditional knockout of CDK12 in the oocyte.

A) Scheme of CDK12 conditionally deleted via the promoter of Zona Pellucida 3 (ZP3)-driven Cre-Lox recombinase in the oocyte. Black arrows indicate the position of the primers for genotyping.

B) PCR for genotyping genomic DNA isolated from different genotypes. Genotyping of progeny for the presence of CDK12($^{tm1c)(flox}$) and Zp3^{-Cre}. The wild-type allele produces a 498-bp product, while the *flox* allele produces a 563-bp product. Zp3^{-Cre} generates a 150 bp product.

C) Immunocytochemical analysis of CDK12 expression and localization in oocytes. Data from three independent biological replicates. CDK12 (red); DAPI (blue); dashed line depicts cell cortex; scale bar 20 µm.

D) Quantification of CDK12 fluorescence intensity in CDK12$^{+/+}$ and CDK12$^{-/-}$ oocytes from (**C**). The number of cells is shown in parentheses. The values from CDK12$^{+/+}$ oocytes were set as 1. Data are presented as mean ± SE; Student's t-test: ***$p < 0.001$.

SI Fig. 2

A

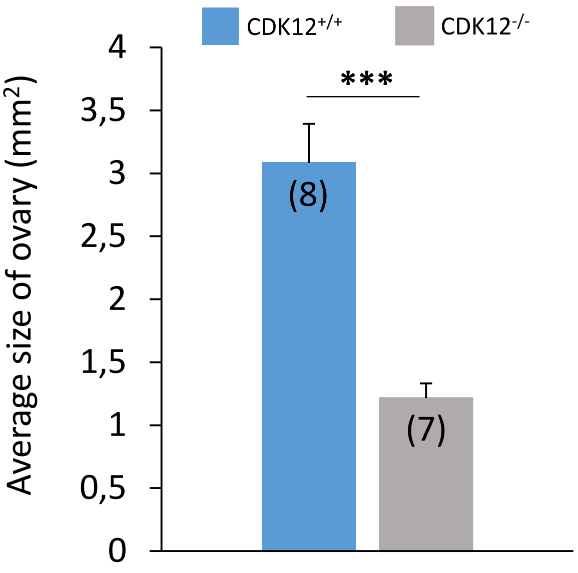

CDK12+/+
CDK12-/-
Average size of ovary (mm2)
4
3,5
3
2,5
2
1,5
1
0,5
0
***
(8)
(7)

B

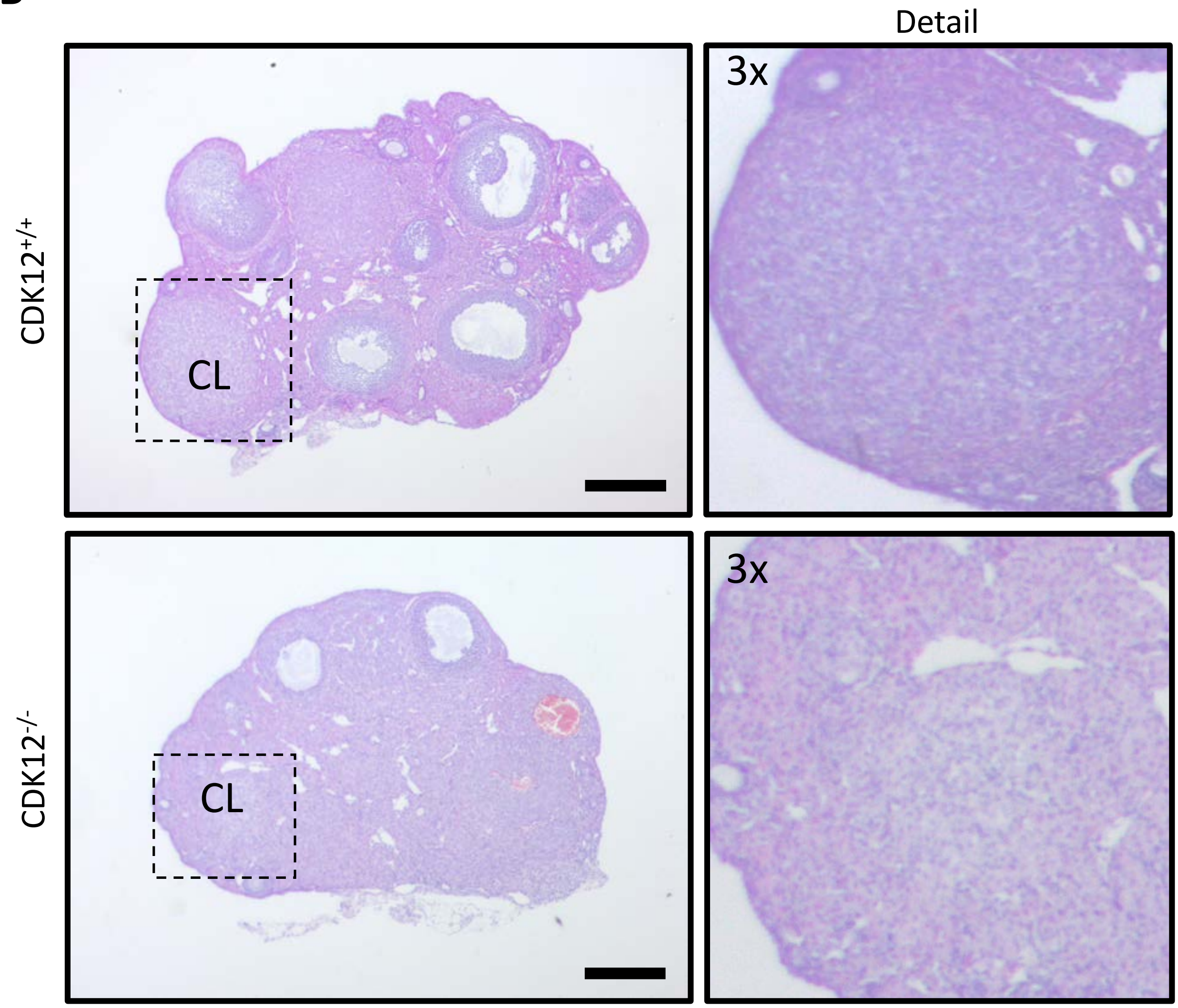

Detail
CDK12+/+
CDK12-/-
CL
CL
3x
3x

SI Fig. 2: The absence of CDK12 in oocytes leads to decreased ovarian size via ceased folliculogenesis.

A) Average size of ovary of $CDK12^{+/+}$ and $CDK12^{-/-}$ females. The number of females for each genotype is shown in parentheses. Data are presented as mean ± SE; Student's t-test: ***$p < 0.001$.

B) The ovary of the $CDK12^{-/-}$ forms a corpus luteum (CL). Representative sections of histologic structures in ovaries of Cdk12-deficient females ($CDK12^{-/-}$) and $CDK12^{+/+}$; scale bar 400 μm.

SI Fig. 3

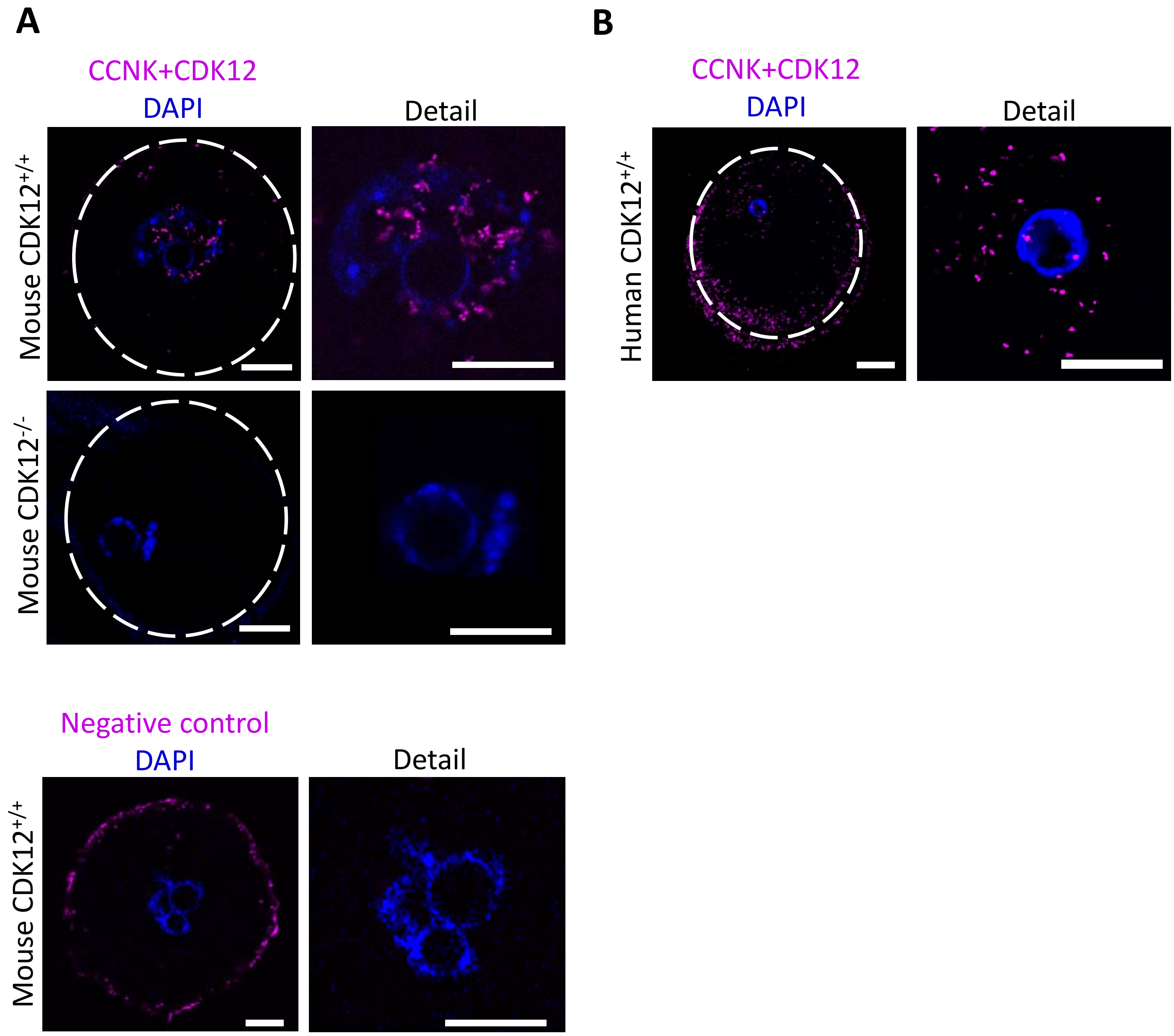

SI Fig. 3: The CDK12/CCNK complex is localized to the nucleus of the oocyte in mice and humans.

A) Representative images of proximity ligation assay showing the nuclear localization and physical interaction of CDK12 and CCNK (purple) in mouse CDK12$^{+/+}$ GV oocytes and no interaction in CDK12$^{-/-}$ oocytes. Detail shows higher magnification of the nuclear region; DAPI (blue); the dashed line shows the cell cortex. Representative images are from three independent biological replicates; n≥28; scale bars 20 µm.

B) Representative images of proximity ligation assay showing the nuclear localization and physical interaction of CDK12 and CCNK (purple) in human oocytes. Detail shows higher magnification of the nuclear region; DAPI (blue); the dashed line shows the cell cortex. Representative images are from three independent biological replicates; n=8; scale bar 20 µm.

SI Fig. 4

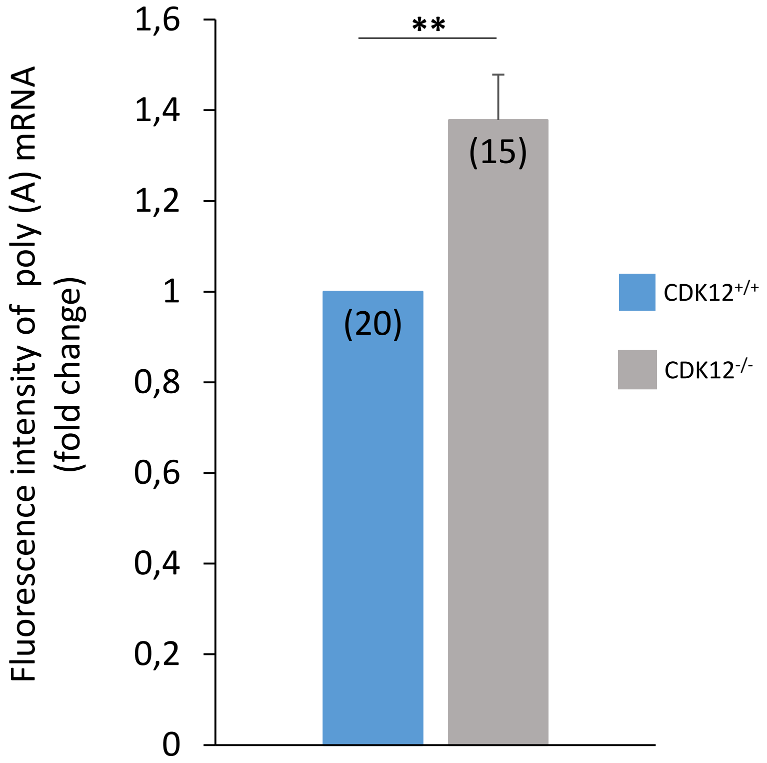

SI Fig. 4: Global poly(A) RNA does not differ between oocytes of different genotypes.

Quantification of poly(A) RNA fluorescence intensity in $CDK12^{+/+}$ and $CDK12^{-/-}$ oocytes from (**Fig. 6D**). The values from $CDK12^{+/+}$ were set as 1. Data from three independent biological replicates. The number of cells shown in parentheses. Data are presented as mean ± SE; Student's *t*-test: **$p < 0.01$.

SI Fig. 5

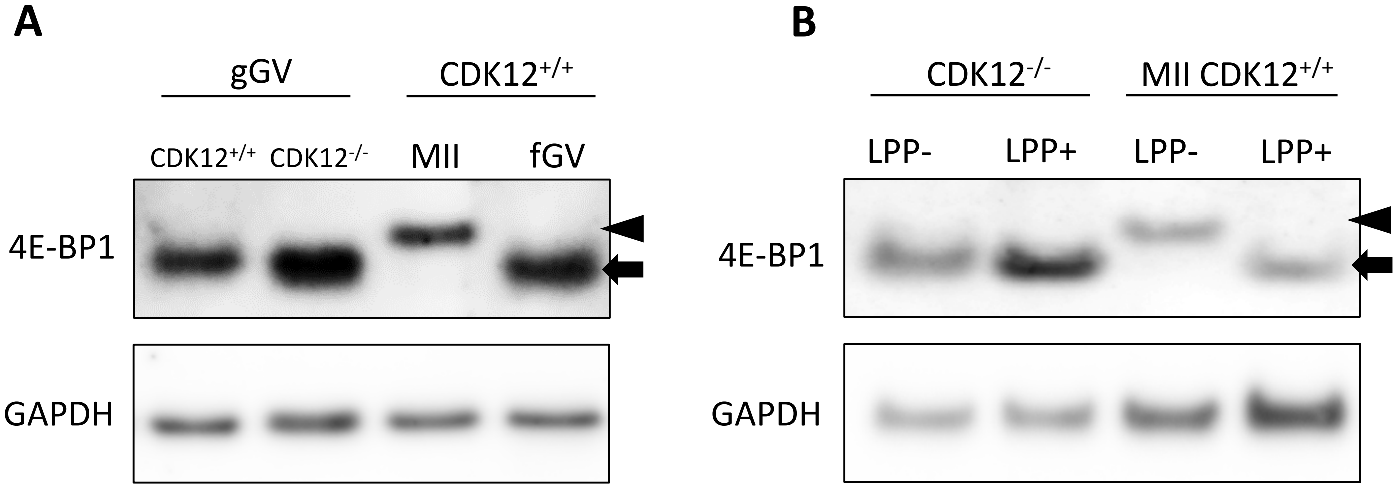

SI Fig. 5: 4E-BP1 is non-phosphorylated in growing oocytes.

A) Western blot analysis of 4E-BP1 expression and phosphorylation shift in growing (gGV) oocytes from different genetic maternal sources. Non-phosphorylated (arrow) and phosphorylated (arrowhead) 4E-BP1. Mature (MII) and fully grown (fGV) oocytes from CDK12$^{+/+}$ were used as controls. GAPDH was used as a loading control. Data were obtained from three biological replicates.

B) Western blot analysis of phosphatase treatment (LPP+) of oocyte samples. Non-phosphorylated (arrow) and phosphorylated (arrowhead) form of 4E-BP1 in growing CDK12$^{-/-}$ oocytes (gGV). Mature (MII) CDK12$^{+/+}$ oocytes were used as a control. GAPDH was used as a loading control. Data are from two biological replicates.

Supplementary Figure

Full immunoblots of segments shown in the main Figure 1A and Figure 5B, E. Arrows denote the bands used.

1A

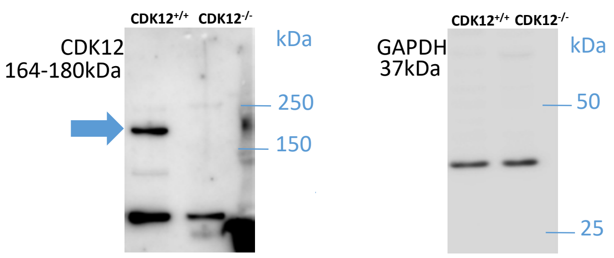

5B

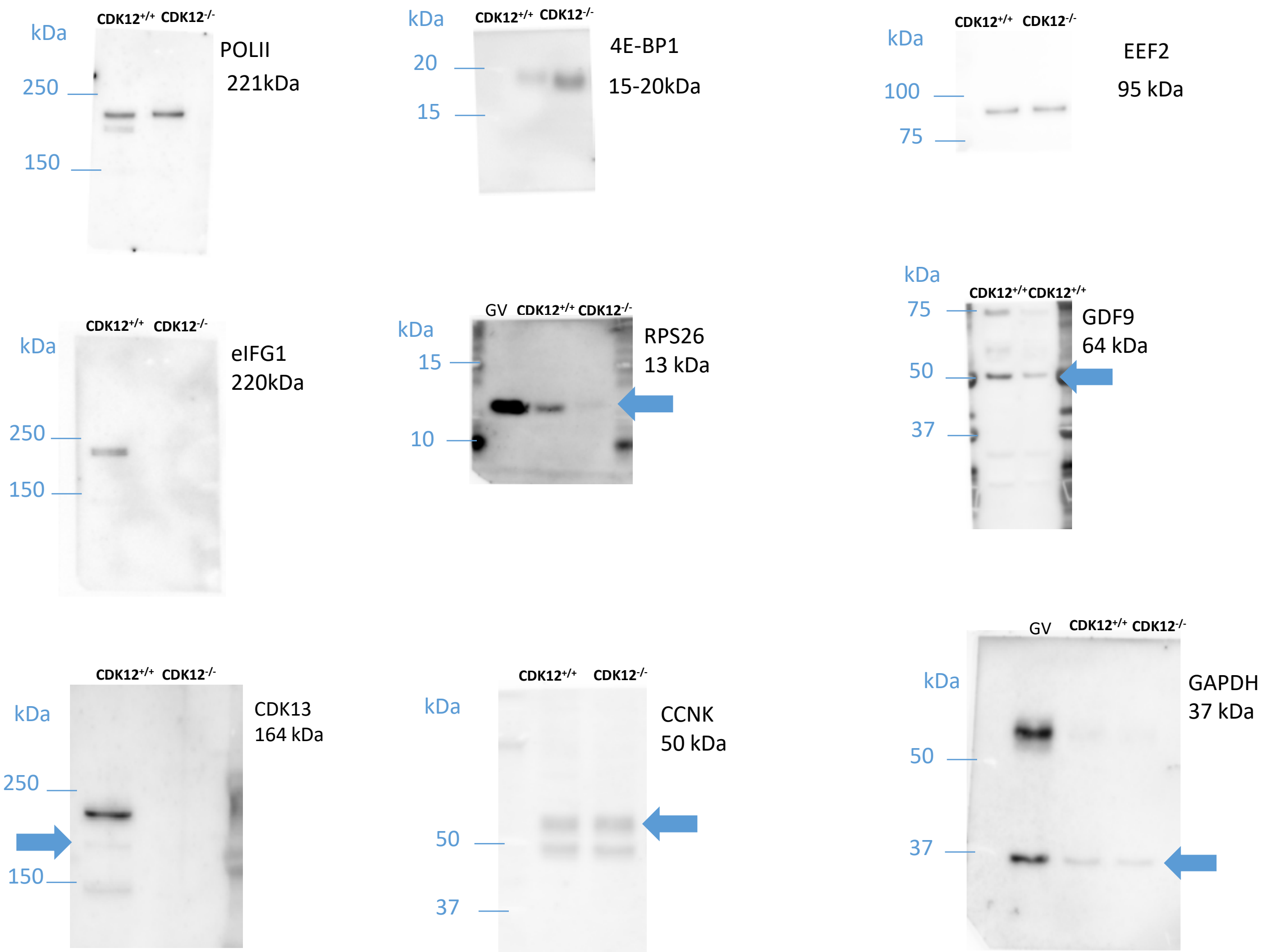

5E

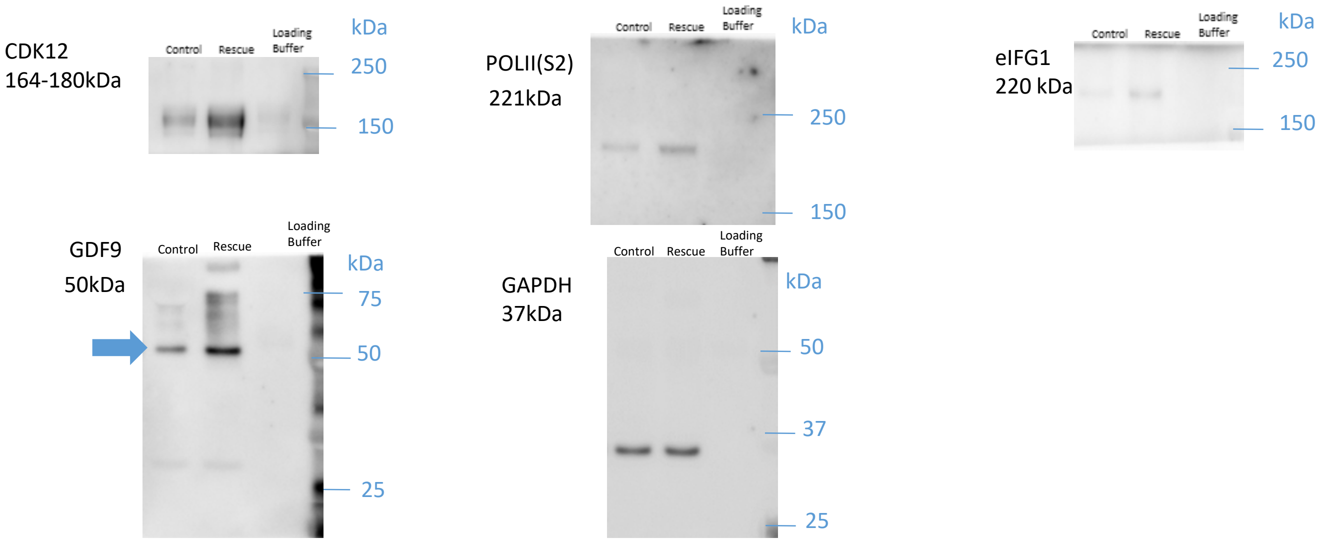

Supplementary Figure Full immunoblots of segments shown in SI.

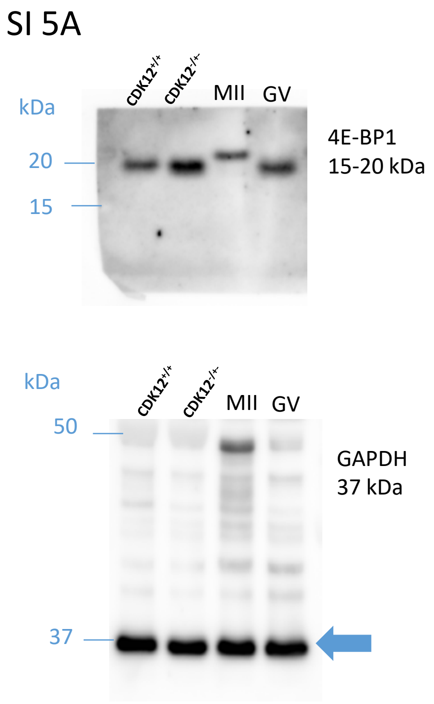

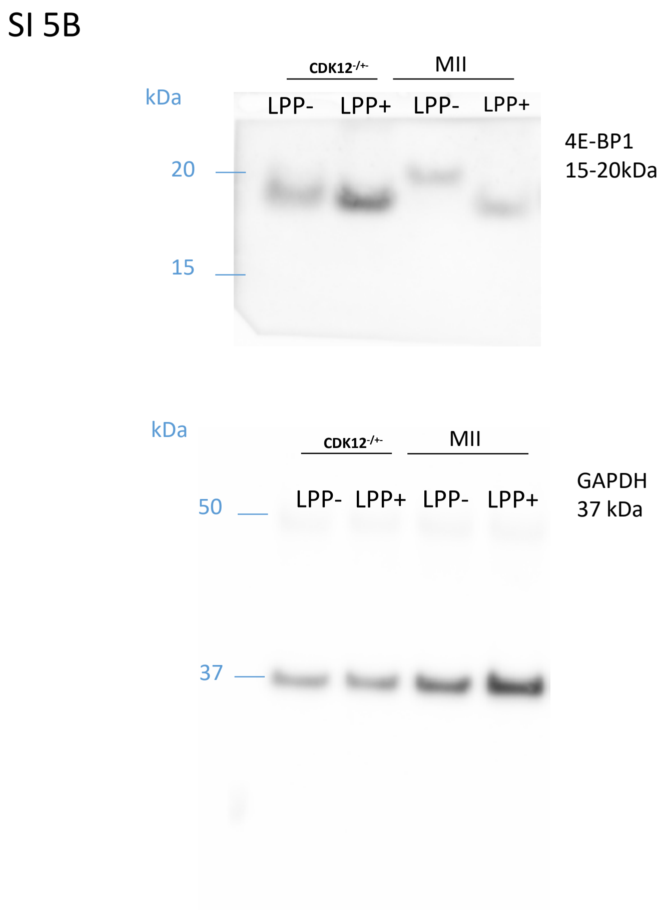

Supplement: Supplementary file 3 — Figures and Legends SI [file 41419_2025_7536_MOESM3_ESM.pdf]
